# Characterizing post-stroke changes in brain microstructure using the mean apparent propagator model

**DOI:** 10.1016/j.nicl.2026.104076

**Published:** 2026-10-21

**Authors:** Ricardo J. Vega, Wesely T. Richerson, Max O. Krucoff, Brian D. Schmit

**Affiliations:** a Department of Biomedical Engineering, Marquette University and Medical College of Wisconsin, Milwaukee, WI, USA; b Department of Neurology, Vanderbilt University Medical Center, Nashville, Tennessee, USA; c Department of Neurosurgery, Medical College of Wisconsin and Froedtert Hospital, Milwaukee, Wisconsin, USA; d Department of Biophysics, Medical College of Wisconsin, Milwaukee, Wisconsin, USA

**Keywords:** Stroke, MAP-MRI, Diffusion tensor imaging, Diffusion MRI, Microstructure, Neuroimaging

## Abstract

This study investigated regional and whole-brain microstructural alterations following stroke using diffusion tensor imaging (DTI) and Mean Apparent Propagator MRI (MAP-MRI). Multi-shell diffusion and T1-weighted MRI data were analyzed from participants with chronic stroke and healthy controls. DTI-derived fractional anisotropy (FA) and mean diffusivity (MD), and MAP-MRI-derived return-to-origin (RTOP), return-to-axis (RTAP), and return-to-plane (RTPP) probabilities were quantified across 13 predefined regions in native diffusion space. Regional group differences were assessed using participant-level median values with Benjamini–Hochberg false discovery rate (BH-FDR) correction across all 65 ROI × diffusion-metric comparisons and age- and sex-adjusted sensitivity analyses. Complementary lesion-relative whole-brain analyses used nonparametric permutation testing with threshold-free cluster enhancement and family-wise error correction.

MAP-MRI demonstrated widespread regional abnormalities, with significant group differences in 11 of 13 regions for RTOP, 12 of 13 for RTAP, and 10 of 13 for RTPP, compared with 8 of 13 for FA and all 13 regions for MD. After adjustment for age and sex, significant differences remained in 11, 10, and 9 regions for RTOP, RTAP, and RTPP, respectively, compared with 6 for FA and all 13 for MD. Whole-brain analyses demonstrated significant corrected abnormalities across all five diffusion metrics, with RTOP and RTAP showing the most spatially extensive Control > Stroke effects, followed by RTPP and FA, whereas MD showed extensive effects in the opposite, Stroke > Control direction. Regional discrimination varied by anatomical region and metric, without a uniform advantage of MAP-MRI over DTI. Exploratory diffusion–function analyses identified several nominal associations, but none survived correction across all comparisons.

These findings demonstrate complementary, region- and metric-specific sensitivity of MAP-MRI and DTI to chronic stroke-related microstructural abnormalities. MAP-MRI identified distributed abnormalities across regional and whole-brain analyses, while MD remained highly sensitive to widespread tissue differences. The differing spatial distributions and regional discriminative performance of MAP-MRI and DTI suggest that these approaches capture partially distinct features of the post-stroke diffusion environment. Together, these findings support their complementary use for characterizing distributed microstructural abnormalities following chronic stroke.

## Highlights

• MAP-MRI provides preliminary evidence of widespread regional microstructural differences after chronic stroke.
• MAP-MRI abnormalities extend beyond the ipsilesional corticospinal tract.
• RTOP and RTAP effects remain robust after age and sex adjustment.
• MAP-MRI abnormalities differ in regional distribution from DTI measures.
• MAP-MRI and DTI provide complementary measures of stroke-related tissue alterations.

## 1 Introduction

Stroke leads to both focal and distributed changes in brain microstructure that extend beyond the infarct core and evolve from acute injury to chronic degeneration. Ischemia triggers a cascade of cellular damage, including axonal injury, Wallerian degeneration, and demyelination, which can disrupt the integrity of white matter tracts (Thomalla et al., 2004; Puig et al., 2010). Beyond the infarct core, remote brain regions connected to the lesion site can exhibit delayed functional and microstructural alterations associated with processes such as secondary degeneration and diaschisis (Feeney & Baron, 1986; Grefkes & Fink, 2011; Carrera & Tononi, 2014). Subcortical structures such as the thalamus and cerebellum are particularly vulnerable to these degenerative processes due to their cortical connections and can exhibit microstructural alterations even in the absence of direct ischemic injury (Kim et al., 2005; Li et al., 2011; Santos et al., 2019). Characterizing these remote abnormalities requires imaging approaches capable of capturing heterogeneous diffusion characteristics across white matter, subcortical gray matter, and cerebellar structures. Accordingly, the present study evaluates whether MAP-MRI can identify chronic stroke-associated diffusion abnormalities in regions that may not be consistently characterized by conventional diffusion tensor imaging (DTI).

DTI has provided important insights into post-stroke white matter damage. DTI-derived metrics such as fractional anisotropy (FA) and mean diffusivity (MD) have been widely used to assess white matter integrity after stroke, and changes in these metrics, particularly within the corticospinal tract, have been associated with axonal damage and motor impairment (Thomalla et al., 2004; Puig et al., 2013; Groisser et al., 2014). However, DTI assumes a Gaussian diffusion process and a single dominant fiber orientation per voxel, limiting its sensitivity in regions characterized by complex fiber architecture or mixed tissue composition (Tournier et al., 2011; Jones et al., 2013). The limitations may be especially relevant in subcortical and remote regions after stroke, where microstructural differences may be subtle, spatially heterogeneous, and difficult to characterize using tensor-based measures alone. Some of these limitations can be addressed using q-space imaging techniques, which characterize the three-dimensional probability distribution of water displacement, providing information about diffusion behavior beyond that captured by the tensor model (Callaghan, 1991; Wedeen et al., 2005). However, most q-space approaches require extended imaging times and high diffusion sampling densities to fully reconstruct the diffusion propagator and associated displacement distribution (Callaghan, 1991; Özarslan & Basser, 2008).

The Mean Apparent Propagator (MAP) MRI technique mitigates many of these issues by modeling the diffusion propagator using a series expansion of Hermite basis functions, which enables estimation of the diffusion propagator using a more practical q-space sampling profile (Özarslan et al., 2013; Fick et al., 2016; Avram et al., 2016). MAP parameters have been shown to be sensitive to brain microstructural changes in silico and in animal models (Avram et al., 2016; Özarslan et al., 2013; Fick et al., 2017). Prior human studies have also demonstrated the feasibility of MAP-MRI for detecting microstructural abnormalities in neurological and systemic disease, including Parkinson's disease and hemodialysis populations (Le et al., 2020; Richerson et al., 2023). More recently, MAP-MRI has been applied to acute ischemic stroke, where MAP-derived measures were evaluated within infarcted tissue and demonstrated relationships with conventional diffusion and neurite-based measures (Diamandi et al., 2025). However, the utility of MAP-MRI for characterizing chronic stroke-related abnormalities beyond the infarct, including within remote subcortical structures such as the thalamus and basal ganglia and within the cerebellum, remains comparatively unexplored. Collectively, these findings suggest that MAP-MRI may provide additional sensitivity to spatially diffuse and heterogeneous microstructural alterations that occur following stroke.

We therefore hypothesized that MAP-MRI would identify chronic stroke-related differences in diffusion characteristics across major white-matter, subcortical, and cerebellar regions that are not consistently captured by conventional DTI measures, with particularly prominent differences expected in subcortical structures and regions with complex tissue architecture.

## 2 Methods

### 2.1 Data collection

#### 2.1.1 Participants

This observational study was a secondary analysis of an existing chronic stroke cohort and included 18 individuals with chronic stroke and 20 healthy controls. The sample size was determined by the number of participants with suitable diffusion MRI data available for the present analyses. Participant demographics are presented in Table 1. All participants provided written informed consent, and study procedures were approved by the Institutional Revie Board at the Medical College of Wisconsin. Participants with stroke were eligible if they were ≥ 6 months post-stroke and able to walk continuously for at least 15 min without assistance. Exclusion criteria included neurological conditions other than stroke, English-speaking status, hearing or visual impairment that would interfere with study procedures, or severe cognitive impairment that would preclude informed consent. Healthy controls were excluded for a history of stroke traumatic brain injury, or other neurological disease or injury; inability to meet walking criterion; or MRI incompatibility. All participants completed MRI safety screening before imaging.

**Table 1:** **Demographic characteristics of study participants.** Age is reported as mean ± standard deviation (SD) and median [interquartile range (IQR)]. Sex is reported as female/male (F/M). Between-group differences in age were evaluated using a two-sided Mann–Whitney *U* test (U = 119.5, *p* = 0.079), and differences in sex distribution were evaluated using Fisher's exact test (OR = 1.875, *p* = 0.516).

Group	Number of Participants (N)	Age, Mean (SD) years	Age, Median (IQR), years	Sex, F/M
Control	20 (12 females)	57.45 (9.42)	56.5 (53.0–64.5)	12/8
Stroke	18 (11 females)	63.06 (8.11)	61.5 (58.0–69.8)	8/10

### 2.2 MRI scans

All images were acquired during a single session using a 3-T MRI (Signa Premier, GE Healthcare, Waukesha, WI, United States) and a 48-channel head coil (GE Healthcare, Waukesha, WI, United States) during a single session. High-resolution T1-weighted anatomical images were acquired using a spoiled gradient-recalled acquisition in steady state (SPGR) pulse sequence (TE = 3.2 ms, TR = 8.16 ms, flip angle = 12°, 256 × 244 matrix, 1 mm^3^ isotropic resolution, field of view = 240 mm, 148 sagittal slices). Diffusion-weighted images were acquired using a q-space sampling scheme optimized for MAP calculations (Olson et al., 2019). Images were acquired in the axial plane with four b = 0 s/mm^2^ images (three anterior-to-posterior phase-encoded, one posterior-to-anterior phase-encoded) and 95 diffusion weighted directions distributed across six b-value shells (TE = 91 ms, TR = 7051 ms, voxel size = 0.86 × 0.86 × 3 mm, 49 slices). The following number of volumes were acquired at each b-value shell: 1000 s/mm^2^, 16 volumes; 2000 s/mm^2^, 3 volumes; 3000 s/mm^2^, 7 volumes; 4000 s/mm^2^, 12 volumes; 5000 s/mm^2^, 18 volumes; 6000 s/mm^2^, 39 volumes. A parallel imaging (ASSET) in-plane acceleration factor of 2 was used, but no multiband imaging was applied.

### 2.3 MRI data processing

#### 2.3.1 Anatomical image processing

The pipeline for image processing is summarized in Fig. 1. T1-weighted images were visually inspected for artifacts, corrected for intensity inhomogeneity using N4 bias correction, brain-extracted using the FMRIB Software Library (FSL) Brain Extraction Tool (BET) and tissue-segmented using FSL's FAST algorithm (Smith, 2002; Zhang et al., 2001). T1-weighted images were registered to the MNI-152 T1 1-mm standard space using Advanced Normalization Tools (ANTs) with rigid (six-parameter), affine, and nonlinear symmetric normalization (SyN) registration (Tustison et al., 2021).

**Fig. 1:**
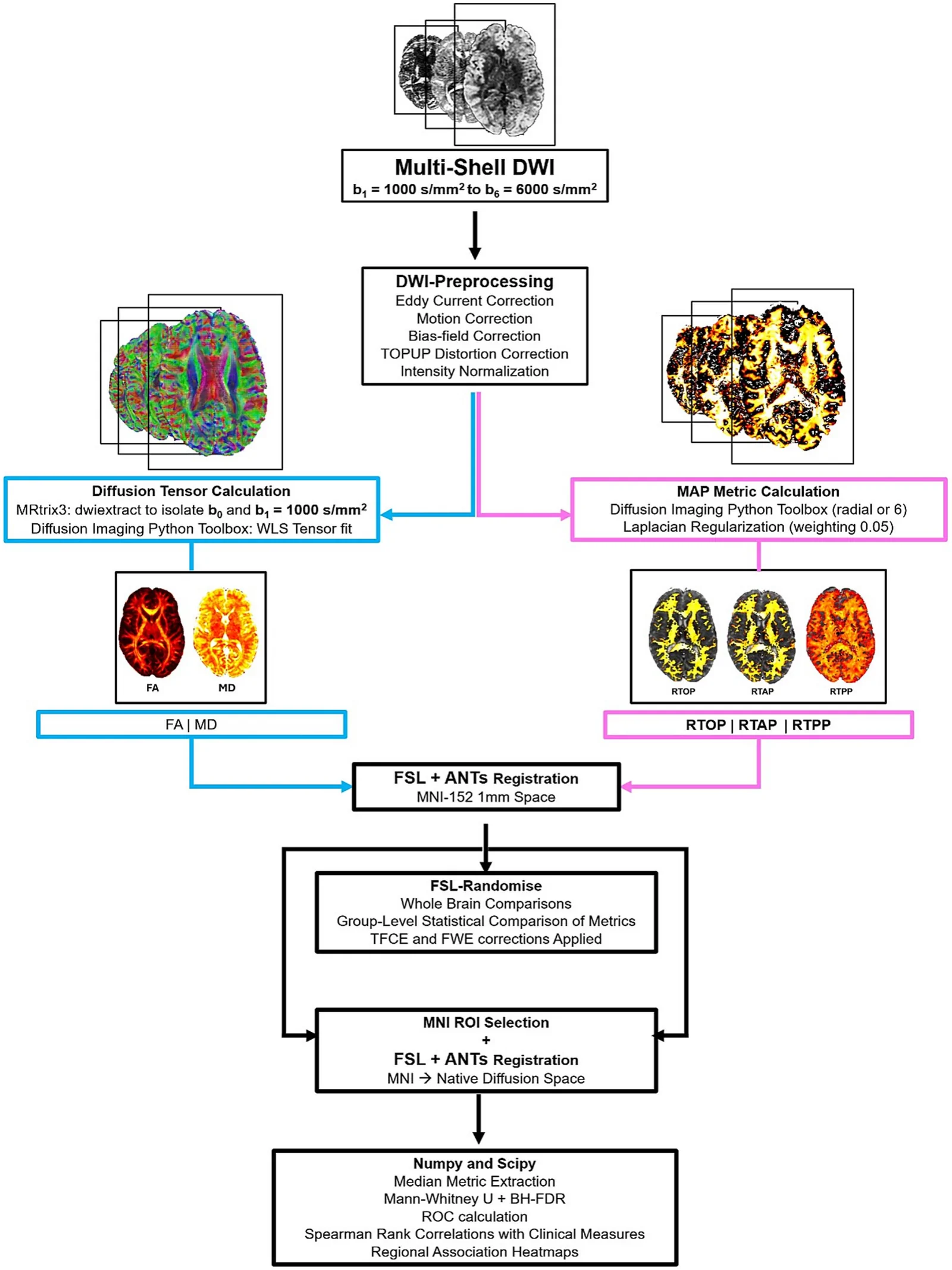
Preprocessing and calculation pipeline for diffusion tensor and mean apparent propagator metrics. Calculations were performed using FSL and python software packages. Group level statistical comparisons were conducted using FSL and region-based analysis was completed using Numpy and Scipy statistical packages

#### 2.3.2 Lesion detection

Preprocessed T1-weighted images from stroke participants were visually inspected to identify lesion location. To supplement visual assessment, lesion identification was also performed using the Lesion Identification with Neighborhood Data Analysis (LINDA) algorithm in R (Pustina et al., 2016). Images from participants with right-hemispheric lesions were flipped across the left-right (x) axis to standardize lesion laterality to the left hemisphere, consistent with LINDA processing framework. LINDA uses a 12-feature classification approach in combination with ANTs to characterize image geometry and participant-specific anatomical abnormalities relative to reference anatomical data (Pustina et al., 2016; Tustison et al., 2021). Resulting lesions masks were registered to the corresponding diffusion and MNI152-1 mm standard spaces for subsequent lesion characterization and spatial comparison with diffusion-derived measures. Lesion distributions are shown in supplementary Fig. S1.

#### 2.3.3 Diffusion image processing

Diffusion-weighted images were preprocessed using the FSL Diffusion Toolbox (FDT). Effective diffusion-weighting values were reconstructed from the encoded gradient table according to the magnitude of each gradient vector, and nonzero diffusion gradient vectors were normalized prior to preprocessing the reconstructed multi-shell acquisition contained 99 volumes distributed across b-0, 1000, 2000, 3000, 4000, 5000, and 6000 s/mm^2^ shells (4, 16, 3, 7, 12, 18, and 39 volumes, respectively). Susceptibility-induced distortions were estimated using FSL TOPUP from b = 0 images obtained from the primary and reverse phase encoding diffusion acquisitions. Acquisition parameters were derived from the corresponding phase-encoding information, and the resulting susceptibility field was incorporated into FSL EDDY processing. EDDY was applied to the full multi-shell diffusion acquisition to correct for eddy-current-induced distortions and participant motion, with outlier replacement enabled. Gradient directions rotated by EDDY were retained for subsequent diffusion-model estimation. Following distortion and motion correction, diffusion-weighted images were denoised using the non-local means algorithm implemented in Diffusion Imaging in Python (DIPY) non-local means algorithm (Coupe et al., 2008; Garyfallidis et al., 2014).

For regional analyses, diffusion-derived maps were retained in participant native diffusion space, with anatomical regions of interest transformed into the corresponding diffusion space for metric extraction. For complimentary whole-brain analyses, diffusion-derived maps were spatially normalized using affine and nonlinear registration procedures implemented in FSL and ANTs and transformed to MNI152-1 mm standard space (Tustison et al., 2021; Jenkinson and Smith, 2001 & Jenkinson et al., 2002; Greve and Fischl, 2009; Andersson et al., 2010).

### 2.4 Calculating diffusion metrics

#### 2.4.1 Diffusion tensor estimation and metrics

Diffusion tensor imaging (DTI) measures were derived from the same preprocessed multi-shell diffusion acquisition used for MAP-MRI modeling; however, tensor fitting used only the b = 0 and b = 1000 s/mm^2^ measurements. This subset comprised four b = 0 volumes and 16 non-collinear diffusion-encoding directions at b = 1000 s/mm^2^. Effective b-values were determined from the encoded gradient table, and nonzero gradient directions were normalized prior to model fitting. Because the symmetric diffusion tensor has six independent elements, the 16 non-collinear diffusion-weighted measurements provided an overdetermined tensor fit, although greater angular sampling can improve the precision of tensor-derived estimates (Basser et al., 1994; Jones, 2004). Diffusion tensors were estimated using weighted least-squares fitting implemented with the DIPY TensorModel (Garyfallidis et al., 2014). Fractional anisotropy (FA) and mean diffusivity (MD) were calculated from the resulting tensor eigenvalues. FA characterized the directional dependence of water diffusion, whereas MD represented the average magnitude of diffusion within the tissue.

#### 2.4.2 MAP modeling and metrics

MAP-MRI modeling was performed on the preprocessed and denoised multi-shell diffusion-weighted images using the MAP-MRI implementation in DIPY (Garyfallidis et al., 2014). Model estimation used a radial order of 6 with Laplacian regularization (weightined =0.05) and a positivity constraint (Fick et al., 2016). From the fitted MAP model, return to the origin probability (RTOP [mm^−3^]), return to axis probability (RTAP [mm^−2^]), and return to plane probability (RTPP [mm^−1^]) were calculated as described in Ozarslan et al., (2013). The metrics characterize complementary properties of the estimated diffusion propagator: RTOP describes the probability density of zero net displacement, while RTAP and RTPP characterize corresponding return probabilities along lower-dimensional projections of the propagator. Because the specific histopathological correlates of these measures in chronic stroke remain uncertain, they were interpreted as diffusion-derived markers of microstructural alteration rather than direct measures of specific tissue components.

### 2.5 Region of interest (ROI) selection

Regions of Interest (ROIs) were selected to compare MAP-MRI and DTI-derived diffusion metrics across white matter, subcortical gray matter, and cerebellar structures relevant to motor function and stroke. Thirteen ROIs were evaluated including the corpus callosum, bilateral corticospinal tracts (CST), bilateral caudate nuclei, bilateral putamen, bilateral thalami, and bilateral cerebellar white and gray matter. The CST and corpus callosum were included as major white matter pathways, while the thalamus, caudate, putamen, and cerebellum were selected because of their involvement in motor function and recovery following stroke and the demonstrated sensitivity of advanced diffusion models to microstructural alterations within subcortical structures (Le et al., 2020; Liu et al., 2020; Schoch et al., 2006; Chen et al., 2019; Park et al., 2011).

Atlas-derived ROI masks were initially defined in MNI152 T1 1-mm standard space. CST and corpus callosum masks were derived from Johns Hopkins University White Matter Tractography Atlas, where ROI probability maps were threshold at 60% to reduce group-wise voxel-wise frequency variability and then binarized (Mori et al., 2005; Wakana et al., 2007; Hua et al., 2008). Subcortical ROIs were obtained by the Harvard-Oxford Subcortical Atlas and the Thalamic Probability Atlas, where probability maps were subjected to the same thresholding and binarization procedures as the white matter ROIs (Makris et al., 2006; Frazier et al., 2005; Desikan et al., 2006; Goldstein et al., 2007). ROIs were transformed into each participants native-diffusion space and binarized. Participant-specific native-space ROIs were subsequently used for extraction of FA, MD, RTOP, RTAP, and RTPP values. This native-space approach minimized interpolation of diffusion-derived metric maps and allowed each participant's diffusion measures to be evaluated within anatomically corresponding ROI masks.

### 2.6 Statistical testing

Participant-specific FA, MD, RTOP, RTAP, and RTPP maps were transformed to MNI152 1-mm standard space for whole-brain voxel-wise analysis. For each metric, group median maps were generated for descriptive visualization, and Control-Stroke difference maps were calculated to characterize the spatial distributions and direction of diffusion alterations between groups. Voxel-wise group differences were assessed using nonparametric permutation testing implemented in FSL randomise with 5000 permutations and threshold-free cluster enhancement (TFCE). Both Control>Stroke and Stroke>Control contrasts were evaluated. The primary model included group as the explanatory variable, with family-wise error (FWE) correction across voxels and statistical significance defined as corrected *p* < 0.05. A sensitivity model additionally included age and sex as covariates. Signed t-statistics were used to indicate the direction and magnitude of group effects within voxels surviving TFCE-FWE correction, and statistical inference was based on the permutation-based TFCE-FWE results.

#### 2.6.1 ROI statistical and discrimination analyses

ROI-based analyses were performed in each participant's native diffusion space using the 13 predefined ROIs. For each participant, the median FA, MD, RTOP, RTAP, and RTPP values were calculated within each ROI and used as the primary regional summary measure. Median values were selected to reduce sensitivity to extreme voxel values and heterogeneous diffusion characteristics within lesioned tissue. Control and stroke groups were compared using two-sided Mann–Whitney *U* tests separately for each diffusion metric and ROI. To account for multiple comparisons across both anatomical regions and diffusion metrics, Benjamini–Hochberg false discovery rate (BH-FDR) correction was applied across the full set of 65 ROI × diffusion-metric comparisons (13 ROIs × 5 diffusion metrics), with statistical significance defined as q < 0.05. Rank-biserial correlation ($r_{\mathrm{r\beta}}$) was calculated as the corresponding nonparametric effect-size measure. ROI mean values were additionally evaluated as a sensitivity analysis to assess whether the observed regional findings were robust to the choice of summary statistic. Regional group differences were additionally evaluated using linear regression models including group, age, and sex as predictors, with HC3 heteroskedasticity-robust standard errors. BH-FDR correction was applied across the full set of 65 ROI × diffusion-metric comparisons independently for the age- and sex-adjusted analyses.

To directly compare the ability of MAP-MRI and DTI metrics to distinguish participants with stroke from healthy controls, receiver operating characteristic (ROC) analyses were additionally performed for each metric and ROI. Discriminative performance was quantified using the area under the ROC curve (AUC), with 95% confidence intervals estimated using 5000 stratified subject-level bootstrap replicates. Differences in AUC between MAP-MRI and DTI metrics were evaluated using paired subject-level bootstrap comparisons, with BH-FDR correction applied across the 13 ROIs separately for each prespecified MAP-MRI–DTI metric comparison. Statistical analyses were implemented in Python using NumPy and SciPy, including scipy.stats.mannwhitneyu for the Mann–Whitney *U* tests.

#### 2.6.2 Lesion-overlap sensitivity analysis and leave-one-out influence analysis

To assess whether regional diffusion differences were attributable to direct infarct involvement, lesion masks were transformed to each participant's native diffusion space and intersected with the final V10 ROI extraction masks. For each ROI, stroke participants whose quantitative lesion mask overlapped the ROI by at least one voxel were excluded, and the Control-versus-Stroke comparison was repeated using the participant-level median diffusion value. Because reliable quantitative lesion masks were available for 13 of 18 stroke participants, the primary sensitivity comparison was made between the valid-lesion-mask baseline and the corresponding non-overlap subset rather than between the full cohort and the non-overlap subset. The five participants with multiple small/microlesions for which reliable quantitative segmentation was unavailable were assigned unknown overlap status and were not classified as non-overlapping. For the non-overlap sensitivity family, Benjamini-Hochberg false discovery rate correction was applied across all 65 ROI x diffusion-metric comparisons, with q < 0.05 considered statistically significant. To assess whether regional group differences were disproportionately influenced by individual stroke participants, a leave-one-out sensitivity analysis was performed. Each of the 65 ROI × diffusion-metric comparisons was repeated after sequentially excluding each stroke participant (18 iterations). Stability was evaluated by examining the direction and magnitude of the rank-biserial effect size across iterations relative to the full-cohort analysis.

#### 2.6.3 Exploratory clinical correlation analysis

Clinical measures were obtained during a separate assessment session a median of 9 days after the MRI session (IQR, 6.5–10.5 days; range, 2–46 days). Measures included the Functional Gait Assessment (FGA; Wrisley et al., 2004), comfortable and fast 10-Meter Walk Tests (10MWT; Collen et al., 1990), and upper- and lower-extremity Fugl-Meyer assessments (Fugl-Meyer et al., 1975). Lower 10MWT completion times indicate faster walking performance, whereas higher FGA and Fugl-Meyer scores indicate better function.

Associations between regional diffusion measures and clinical function were assessed within the stroke group using Spearman rank correlations. Subject-level median FA, MD, RTOP, RTAP, and RTPP values from each of the 13 native-space ROIs were correlated with FGA, comfortable and fast 10MWT performance, and upper- and lower-extremity Fugl-Meyer scores. Participants with missing clinical data were excluded separately for each outcome. To account for multiple testing, BH-FDR correction was applied across the 65 planned correlations (13 ROIs × 5 diffusion metrics) separately within each clinical outcome, with statistical significance defined as q < 0.05. Spearman's rho (ρ) was reported to characterize the strength and direction of each association.

#### 2.6.4 Retrospective power and sensitivity analysis

To provide quantitative context for the available sample size, retrospective power was estimated for the primary median-based Control-versus-Stroke ROI comparisons using the observed rank-biserial effect sizes and ROI-specific sample sizes. Approximate power for the two-sided Mann–Whitney *U* test was estimated using Monte Carlo simulation, and minimum detectable effects corresponding to 80% power were calculated. These analyses were considered descriptive and were not used to determine statistical significance or reinterpret nonsignificant findings.

#### 2.6.5 Descriptive lesion location analysis

To provide exploratory context regarding lesion location, Stroke participants were categorized as having cortical, subcortical, or multifocal infarcts. Median and interquartile-range diffusion measures were summarized within each subgroup. Because subgroup sizes were small and unequal and the study was not powered for lesion-location comparisons, no inferential subgroup testing was performed.

## 3 Results

### 3.1 Participant characteristics

Eighteen participants with chronic stroke and 20 healthy controls were included. Median Age was 61.5 years [IQR, 58.0–69.8] in the stroke group and 56.5 years [53.0–64.5] in the control group. Age did not significantly differ between groups (Mann-Whitney U = 119.5, *p =* 0.079*)*, nor did sex distribution (Fischer's exact OR = 1.875, *p* = 0.516); Table 1.

### 3.2 Regional differences in MAP-MRI and DTI metrics

Primary native-space ROI analyses using subject-level median values demonstrated widespread differences in diffusion measures between participants with chronic stroke and healthy controls (Fig. 2, Fig. 3; Table 2). Following BH-FDR correction across all 65 ROI × diffusion-metric comparisons, significant group differences were identified in 11 of 13 ROIs for RTOP, 12 of 13 for RTAP, and 10 of 13 for RTPP. FA demonstrated a more restricted pattern, with significant differences in 8 of 13 ROIs, whereas MD differed significantly in all 13 ROIs.

**Fig. 2:**
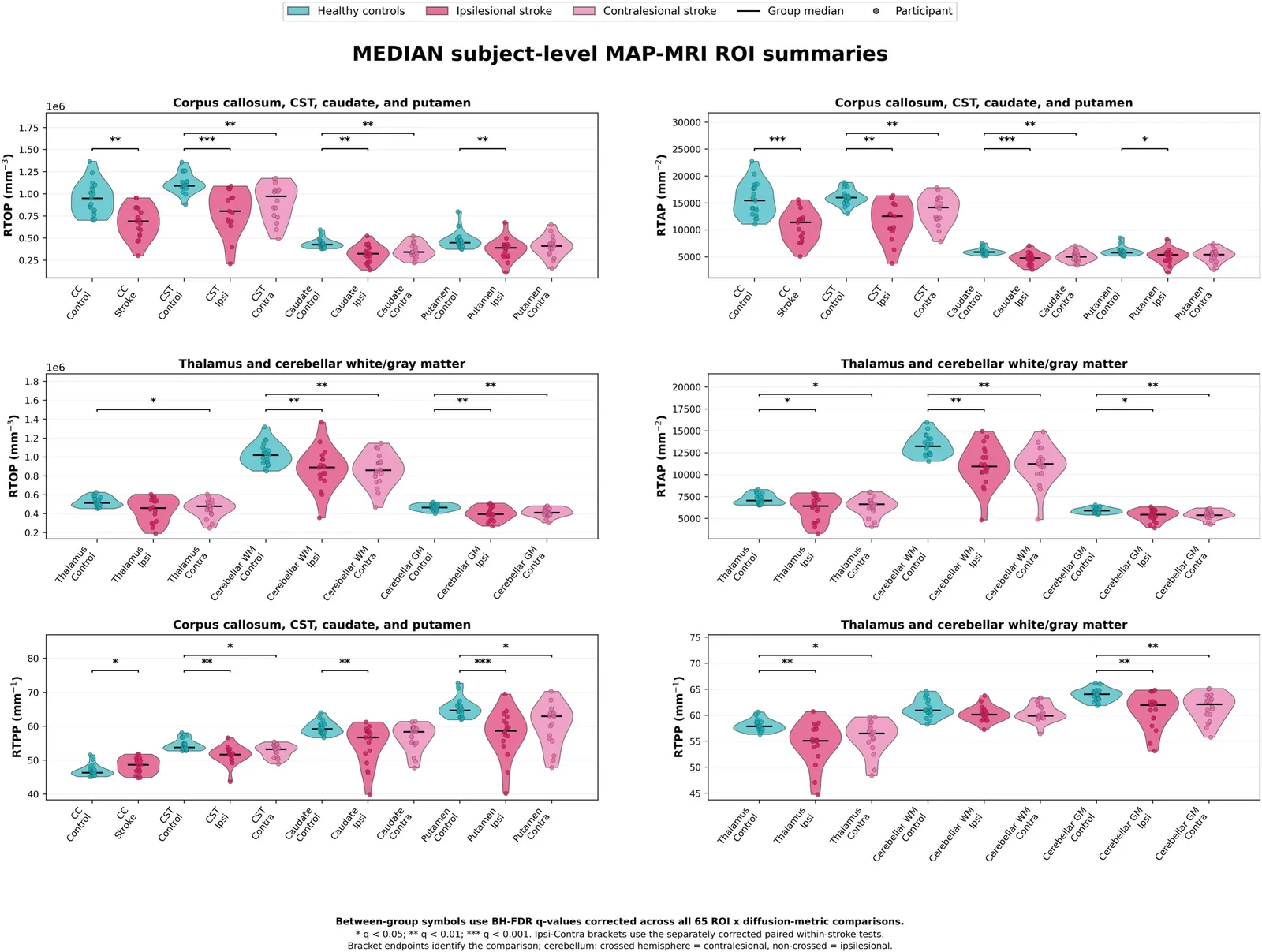
Regional differences in MAP-MRI and DTI diffusion 352 metrics between participants with chronic stroke and healthy controls. Violin plots show the distributions of subject-level native space ROI median values for return-to-origin probability (RTOP), return-to-axis probability (RTAP), and return-to-plane probability (RTPP), across the corpus callosum, bilateral cortical spinal tracts (CST), caudate, putamen, thalamus, and cerebellar white and gray matter. Individual observations are overlaid on each distribution; white diamonds indicate the group medians. Between-group significance was assessed using two-sided Mann–Whitney U tests with BH-FDR correction across all 65 ROI × diffusion-metric comparisons. Ipsilesional–contralesional brackets indicate separately corrected paired comparisons, *q<0.05, **q <0.01, **q <0.001. *Bracket endpoints identify the comparison; cerebellum: crossed hemisphere = contralesional, non-crossed = ipsilesional.*

**Fig. 3:**
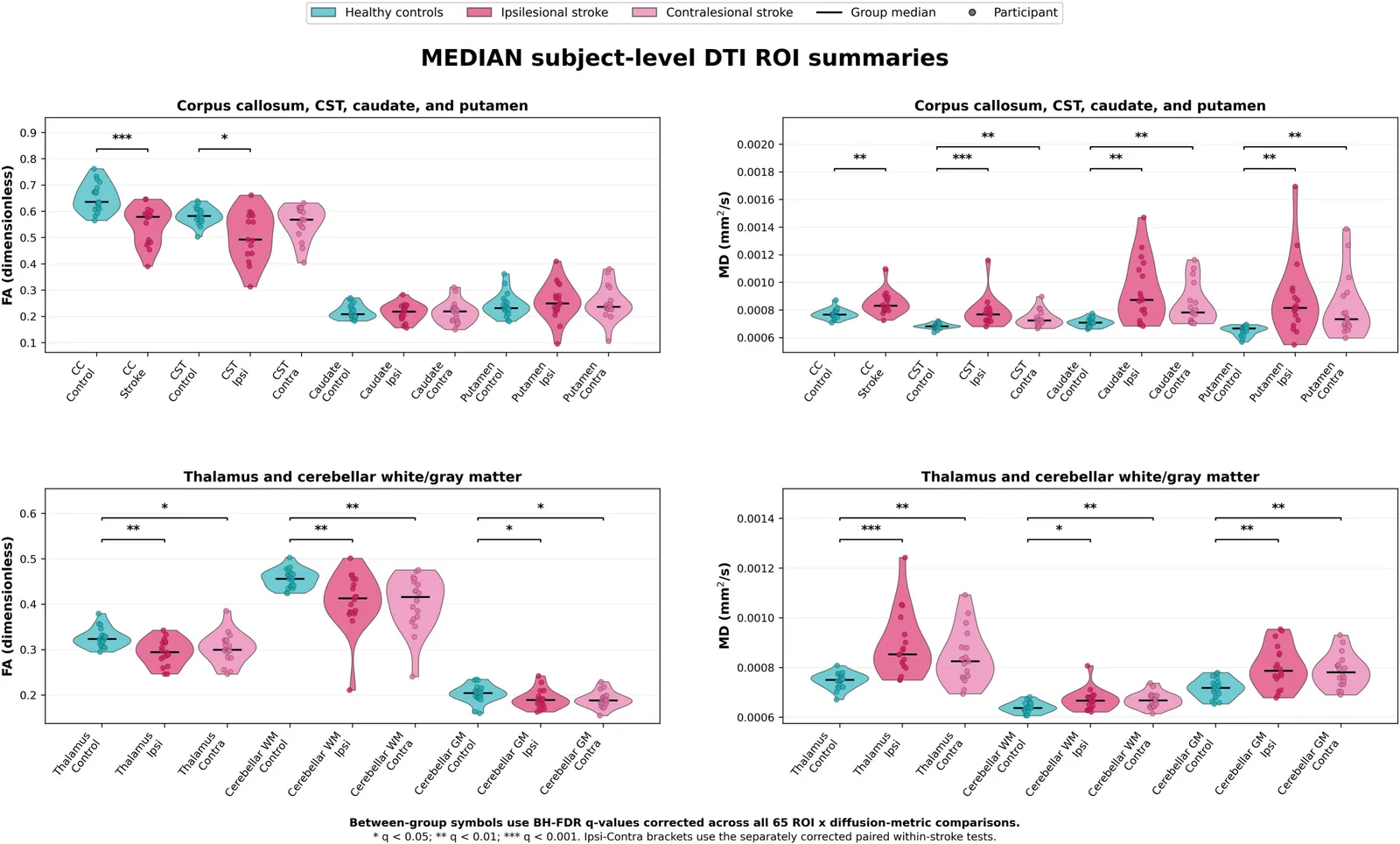
Regional differences in DTI diffusion metrics between 363 participants with chronic stroke and healthy controls. Violin plots show the distributions of subject-level native space ROI median values for Fractional Anisotropy (FA) and mean diffusivity (MD), across the corpus callosum, bilateral cortical spinal tracts (CST), caudate, putamen, thalamus, and cerebellar white and gray matter. Individual observations are overlaid on each distribution; white diamonds indicate the group medians. Control and stroke groups are shown in teal and magenta, respectively. Between-group significance was assessed using two-sided Mann–Whitney U tests with BH-FDR correction across all 65 ROI × diffusion-metric comparisons. Ipsilesional–contralesional brackets indicate separately corrected paired comparisons, *q < 0.05, **q < 0.01, **q < .001. *Bracket endpoints identify the comparison; cerebellum: crossed hemisphere = contralesional, non-crossed = ipsilesional.*

**Table 2:** **Regional differences in diffusion MRI measures between control and stroke.** Cells show rank-biserial correlation, $r_{\mathrm{r\beta}}$, and BH-FDR-adjusted q-values. BH-FDR correction was applied across all 65 ROI × diffusion-metric comparisons (13 ROIs × 5 diffusion metrics). Positive $r_{\mathrm{r\beta}}$ indicates Control > Stroke; Negative $r_{\mathrm{r\beta}}$ indicates Stroke>Control. Bold = q < 0.05.

ROI	RTOP	RTAP	RTPP	FA	MD
Corpus callosum	+0.72 q = 0.001**	+0.76 q ≤0.001***	−0.41 q = 0.044*	+0.78 q ≤0.001***	−0.73 q = 0.001**
CST Ipsi	+0.85 q ≤0.001***	+0.74 q = 0.001**	+0.76 q = 0.001**	+0.52 q = 0.017*	−0.89 q ≤0.001***
CST Contra	+0.62 q = 0.005**	+0.57 q = 0.008**	+0.49 q = 0.021*	+0.19 q = 0.362	−0.62 q = 0.005**
Caudate Ipsi	+0.70 q = 0.001**	+0.78 q ≤0.001***	+0.57 q = 0.007**	+0.01 q = 0.975	−0.72 q = 0.001**
Caudate Contra	+0.55 q = 0.009**	+0.57 q = 0.007**	+0.37 q = 0.072	+0.10 q = 0.622	−0.68 q = 0.002**
Putamen Ipsi	+0.56 q = 0.008**	+0.45 q = 0.029*	+0.78 q ≤0.001***	−0.21 q = 0.315	**−0.73** q = 0.001**
Putamen Contra	+0.36 q = 0.078	+0.36 q = 0.078	+0.54 q = 0.010*	−0.13 q = 0.543	**−0.68** q = 0.002**
Thalamus Ipsi	+0.36 q = 0.078	+0.42 q = 0.041*	+0.60 q = 0.005**	+0.62 q = 0.005**	**−0.83** q ≤0.001***
Thalamus Contra	+0.46 q = 0.026*	+0.46 q = 0.027*	+0.44 q = 0.031*	+0.47 q = 0.024*	**−0.59** q = 0.005**
CB-WM Ipsi	+0.54 q = 0.008**	+0.63 q = 0.004**	+0.36 q = 0.072	+0.60 q = 0.005**	**−0.50** q = 0.015*
CB-WM Contra	+0.56 q = 0.007**	+0.71 q = 0.001**	+0.31 q = 0.120	+0.60 q = 0.005**	**−0.61** q = 0.005**
CB-GM Ipsi	+0.58 q = 0.006**	+0.51 q = 0.013*	+0.58 q = 0.005**	+0.43 q = 0.032*	**−0.62** q = 0.004**
CB-GM Contra	+0.62 q = 0.004**	+0.55 q = 0.008**	+0.58 q = 0.005**	+0.49 q = 0.018*	**−0.63** q = 0.004**

MAP-MRI abnormalities were distributed across major white-matter, subcortical, and cerebellar regions. RTOP was significantly lower in stroke in 11 of 13 regions, with the contralesional putamen and ipsilesional thalamus not surviving BH-FDR correction. RTAP demonstrated the broadest MAP-MRI pattern, with significant reductions in all regions except the contralesional putamen. RTPP differences were significant in 10 of 13 regions, with the contralesional caudate and bilateral cerebellar white matter not surviving correction. Particularly large effects were observed within ipsilesional motor and subcortical regions. In the ipsilesional CST, rank-biserial effect sizes were + 0.85 for RTOP (q < 0.001), +0.74 for RTAP (q < 0.001), and + 0.76 for RTPP (q = 0.001), indicating substantially lower MAP-MRI values in stroke. The ipsilesional thalamus demonstrated significant effects for RTAP (+0.42, q = 0.041) and RTPP (+0.60, q = 0.005), whereas the RTOP effect (+0.36, q = 0.078) did not survive BH-FDR correction. Strong MAP-MRI effects were also observed in the ipsilesional caudate and putamen, corpus callosum, and bilateral cerebellar gray matter, with RTOP and RTAP additionally demonstrating significant abnormalities in bilateral cerebellar white matter.

DTI measures demonstrated a distinct regional pattern. FA was significantly lower in stroke within the corpus callosum, ipsilesional CST, bilateral thalamus, bilateral cerebellar white matter, and bilateral cerebellar gray matter. The largest FA effect was observed in the corpus callosum ($r_{\mathrm{r\beta}}$= = + 0.78, q < 0.001), followed by the ipsilesional thalamus ($r_{\mathrm{r\beta}}$= + 0.62, q = 0.005). In contrast, MD was significantly higher in stroke across all 13 ROIs, with particularly large effects in the ipsilesional CST ($r_{\mathrm{r\beta}}$= − 0.89, q < 0.001), ipsilesional thalamus ($r_{\mathrm{r\beta}}$= − 0.83, q < 0.001), corpus callosum ($r_{\mathrm{r\beta}}$= − 0.73, q < 0.001), and ipsilesional putamen ($r_{\mathrm{r\beta}}$= − 0.73, q < 0.001). Collectively, these findings demonstrate complementary regional sensitivity of MAP-MRI and DTI measures. MAP-MRI identified robust abnormalities across most evaluated regions and showed broader regional sensitivity than FA, whereas MD demonstrated the most spatially uniform pattern of group differences.

### 3.3 *Robustness to age, sex Adjustmen*t, and lesion overlap

Regional group differences were largely preserved after adjustment for age and sex, although the number of FDR-significant ROIs decreased for several metrics (Table 3). Significant group differences remained in 11 of 13 ROIs for RTOP, 10 of 13 for RTAP, 9 of 13 for RTPP, 6 of 13 for FA, and all 13 ROIs for MD. Adjusted FA effects remained significant in the corpus callosum, ipsilesional CST, bilateral thalamus, and bilateral cerebellar white matter, whereas MD remained significant across all evaluated ROIs. Overall, the regional findings were robust to demographic adjustment, with persistent effects across multiple MAP-MRI and DTI measures.

**Table 3:** **Robustness of primary ROI findings after adjustment for age and sex.** Adjusted models used ROI median = Group + Age + Sex with HC3 robust standard errors. BH-FDR correction was applied across all 65 ROI × diffusion-metric comparisons (13 ROIs × 5 diffusion metrics), separately for the primary and age- and sex-adjusted analyses. Abbreviations: CC, corpus callosum; Cau, caudate; Put, putamen; Thal, thalamus; CB-WM, cerebellar white matter; CB-GM, cerebellar gray matter.

Metric	Primary q < 0.05	Adjusted q < 0.05	Adjusted FDR-significant ROIs
RTOP	11/13	11/13	CC, CST-Ipsi, CST-Contra, Cau-Ipsi, Cau-Contra, Put-Ipsi, Thal-Ipsi, Thal-Contra, CB-WM-Contra, CB-GM-Ipsi, CB-GM-Contra
RTAP	12/13	10/13	CC, CST-Ipsi, Cau-Ipsi, Cau-Contra, Thal-Ipsi, Thal-Contra, CB-WM-Ipsi, CB-WM-Contra, CB-GM-Ipsi, CB-GM-Contra
RTPP	10/13	9/13	CST-Ipsi, CST-Contra, Cau-Ipsi, Cau-Contra, Put-Ipsi, Put-Contra, Thal-Ipsi, CB-GM-Ipsi, CB-GM-Contra
FA	8/13	6/13	CC, CST-Ipsi, Thal-Ipsi, Thal-Contra, CB-WM-Ipsi, CB-WM-Contra
MD	13/13	13/13	CC, CST-Ipsi, CST-Contra, Cau-Ipsi, Cau-Contra, Put-Ipsi, Put-Contra, Thal-Ipsi, Thal-Contra, CB-WM-Ipsi, CB-WM-Contra, CB-GM-Ipsi, CB-GM-Contra

Quantitative lesion masks were available for 13 of 18 stroke participants. After excluding participants whose lesion directly overlapped the ROI under study, significant Control-versus-Stroke differences remained in 4/13 RTOP, 4/13 RTAP, 3/13 RTPP, 3/13 FA, and 10/13 MD regional comparisons after global 65-test BH-FDR correction. The number of significant comparisons was reduced relative to the valid-mask baseline (9/13 RTOP, 6/13 RTAP, 7/13 RTPP, 4/13 FA, and 12/13 MD), indicating that direct lesion involvement contributed to a subset of the regional abnormalities. However, significant differences persisted after direct-overlap exclusion for all five diffusion metrics, supporting the presence of regional abnormalities that were not solely attributable to directly infarcted tissue. Exact ROI-specific retained sample sizes and effect sizes are reported in Supplementary Table S5.

All 54 ROI × diffusion-metric comparisons that were significant in the primary analysis retained the same direction of effect across all 18 leave-one-out iterations, with no direction reversals. Across all 65 comparisons, 64/65 showed complete directional stability; the only directionally variable comparison was ipsilesional caudate FA, which was non-significant in the full-cohort analysis (rrb = 0.009, q = 0.975). Most significant findings also remained below the global BH-FDR threshold across all iterations, although three borderline comparisons showed greater q-value sensitivity to individual participant exclusion. Overall, these results indicate that the principal regional group differences were not attributable to any single stroke participant (Supplementary Table S3).

### 3.4 Regional discrimination between stroke and control participants

ROC analysis demonstrated substantial but regionally heterogeneous discrimination between participants with stroke and healthy controls (Fig. 4, Fig. 5). Absolute AUC values were high across several motor, subcortical, and cerebellar regions, although the metric providing the greatest discrimination varied by region.

**Fig. 4:**
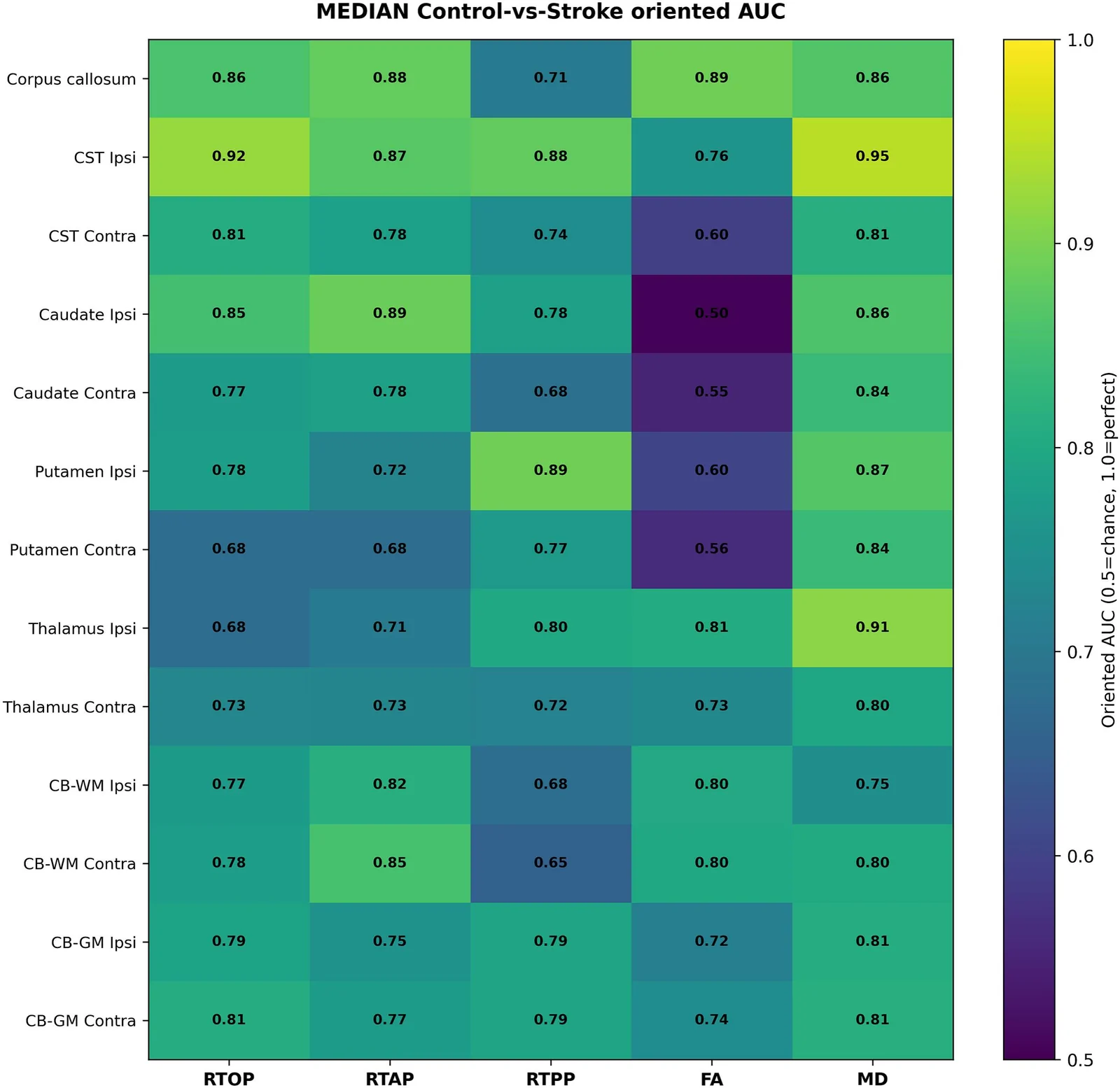
Regional discriminative performance of MAP-MRI and DTI diffusion metrics between participants with chronic stroke and healthy controls. The heatmap displays area under the receiver operating characteristic curve (AUC) for subject-level median return-to-origin probability (RTOP), return-to-axis probability (RTAP), return-to-plane probability (RTPP), Fractional Anisotropy (FA), mean diffusivity across 13 lesion-relative regions of interest. AUC values were oriented such that values approaching 1.0 indicate greater discrimination between groups irrespective of the direction of underlying diffusion abnormality, whereas values approaching 0.5 indicate chance level discrimination.

**Fig. 5:**
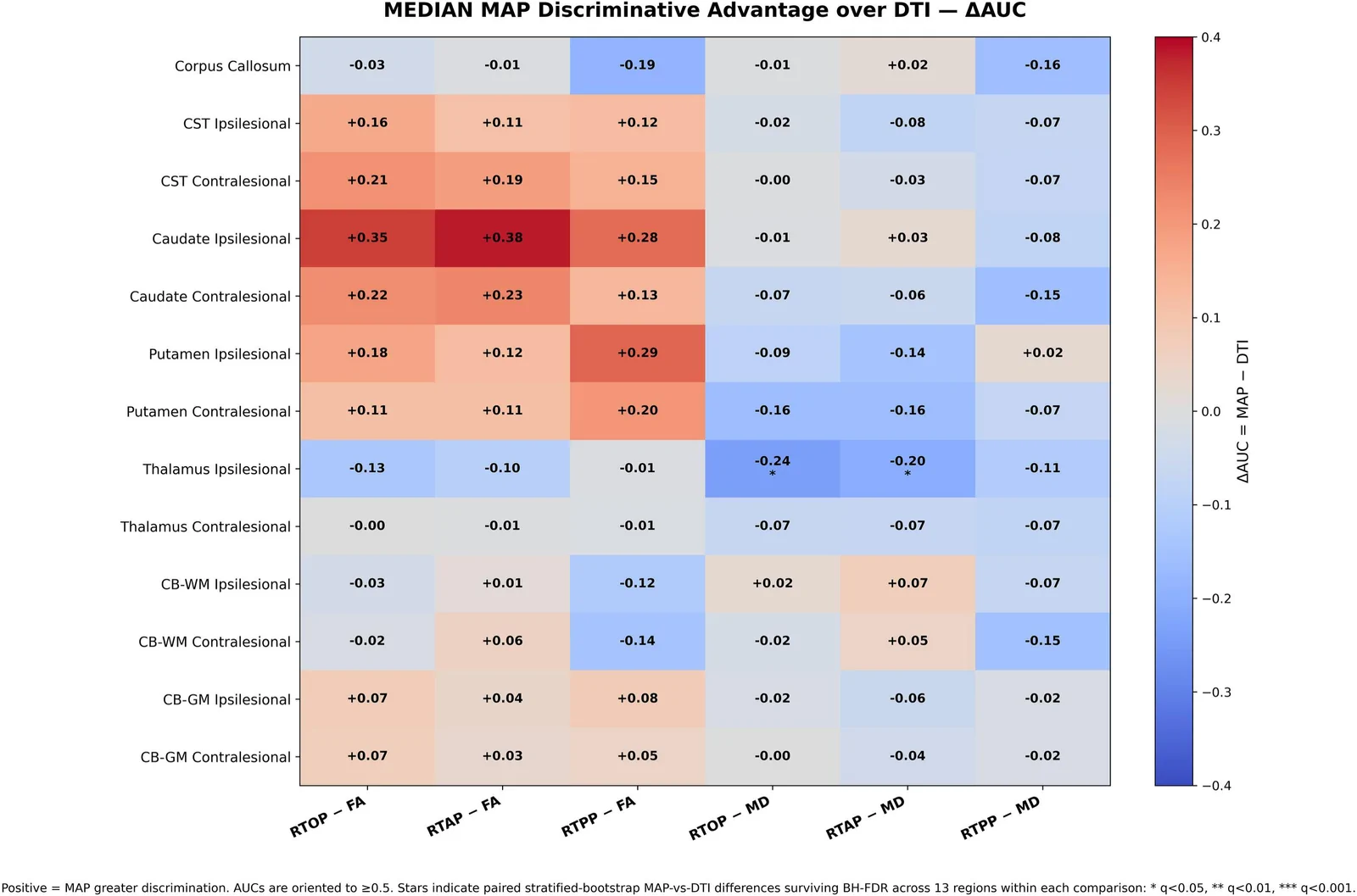
Paired regional comparison of discriminative performance 458 between MAP-MRI and DTI diffusion metrics. The heatmap displays differences in AUC (Δ*AUC*) between each MAP-Metric (RTOP, RTAP, and RTPP) and conventional DTI metrics (FA and MD) across 13 lesion-relative regions of interest. Positive ΔAUC values indicate greater discrimination by the MAP-MRI metric, whereas negative values favor corresponding DTI metric. Statistical comparisons were performed using 5,000 paired subject-level bootstrap replicates, with Benjamini-Hochberg false discovery rate (BH-FDR) correction across the 13 separately for each MAP-DTI comparison. *Positive = MAP greater discrimination. AUCs are oriented to* ≥ 0.5, stars indicate paired stratified-bootstrap MAP-vs-DTI differences surviving BH-FDR across 13 regions within each comparison *q < 0.05, **q < 0.01, **q 467 ***<**.001.*

The strongest discrimination was observed within the ipsilesional CST, where MD achieved an AUC of 0.95, followed by RTOP (AUC = 0.92), RTPP (AUC = 0.88), RTAP (AUC = 0.87), and FA (AUC = 0.76). The ipsilesional thalamus also demonstrated strong discrimination, particularly for MD (AUC = 0.91) and FA (AUC = 0.81), followed by RTPP (AUC = 0.80), RTAP (AUC = 0.71), and RTOP (AUC = 0.68). MAP-MRI additionally demonstrated moderate-to-strong discrimination in cerebellar gray matter, with RTOP, RTAP, and RTPP AUCs of 0.79, 0.75, and 0.79 ipsilesionally and 0.81, 0.77, and 0.79 contralesionally, respectively.

Paired analyses further demonstrated that the relative discriminative performance of MAP-MRI and DTI was region dependent (Fig. 5). The largest positive AUC differences between MAP-MRI measures and FA were observed in the caudate, particularly ipsilesionally, whereas differences between MAP-MRI and MD were generally smaller and frequently favored MD. Significant differences favoring MD were observed in the ipsilesional thalamus for RTOP and RTAP. Collectively, these findings indicate that discrimination was not consistently dominated by a single diffusion model; rather, MAP-MRI and DTI demonstrated region- and metric-dependent differences in discrimination between participants with stroke and healthy controls.

### 3.5 Whole-brain spatial distribution of diffusion abnormalities

Whole-brain voxel-wise analyses demonstrated spatially distributed group differences across diffusion metrics after adjustment for age and sex (Fig. 6). Statistical inference was based on nonparametric permutation testing with threshold-free cluster enhancement (TFCE) and whole-brain family-wise error (FWE) correction (pFWE <0.05). Results were displayed in lesion-relative orientation, with ipsilesional and contralesional hemispheres aligned across participants.

**Fig. 6:**
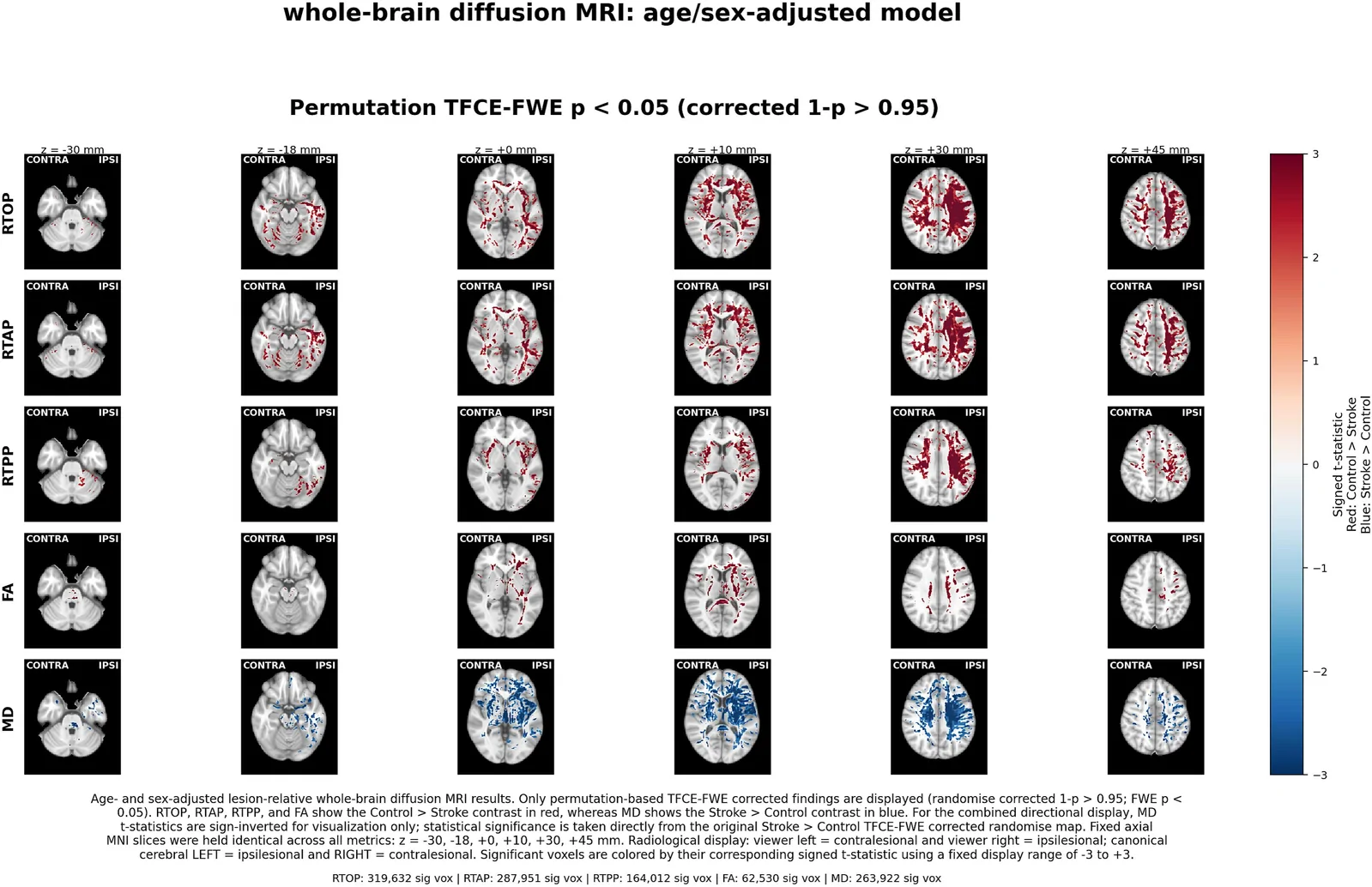
Age- and sex-adjusted lesion-relative whole-brain diffusion MRI group differences. Permutation-based TFCE results surviving whole-brain FWE correction (pFWE < 0.05) are shown for RTOP, RTAP, RTPP, FA, and MD. Fixed axial MNI slices (z = −30, −18, 0, +10, +30, and +45 mm) are displayed in radiological convention, with viewer left representing the contralesional hemisphere and viewer right representing the ipsilesional hemisphere. Red indicates significant Control > Stroke effects for RTOP, RTAP, RTPP, and FA, whereas blue indicates significant Stroke > Control effects for MD. Significant voxels are colored according to the corresponding signed t-statistic using a common display range of −3 to +3. *Age- and sex-adjusted lesion-relative whole-brain diffusion MRI results. Only permutation-based TFCE-FWE corrected findings are displayed (randomise corrected 1-p > 0.95; FWE p < 0.05). RTOP, RTAP, RTPP, and FA show the Control > Stroke contrast in red, whereas MD shows the Stroke > Control contrast in blue. For the combined directional display, MD t-statistics are sign-inverted for visualization only; statistical significance is taken directly from the original Stroke > Control TFCE-FWE corrected randomise map. Fixed axial MNI slices were held identical across all metrics: z = -30, -18, +0, +10, +30, +45 mm. Radiological display: viewer left = contralesional and viewer right = ipsilesional; canonical cerebral LEFT = ipsilesional and RIGHT = contralesional. Significant voxels are colored by their corresponding signed t-statistic using a fixed display range of - 3 to + 3. **RTOP: 319,632 sig vox | RTAP: 287,951 sig vox | RTPP: 164,012 sig vox | FA: 62,530 sig vox | MD: 263,922 sig vox***

Significant Control > Stroke effects were observed for all three MAP-MRI measures and FA. RTOP demonstrated the most spatially extensive Control > Stroke effects (319,632 significant voxels), followed by RTAP (287,951 voxels), RTPP (164,012 voxels), and FA (62,530 voxels). These differences were distributed across multiple anatomical levels and involved both ipsilesional and contralesional territories. In contrast, MD demonstrated extensive Stroke > Control effects (263,922 significant voxels), with no significant effects in the opposite direction. For visualization in Fig. 6, MD t-statistics were sign-inverted so that the Stroke > Control effect could be displayed using the same directional color convention as the other diffusion metrics; this transformation was applied for display purposes only and did not alter the statistical inference or direction of the underlying MD effect. Collectively, these findings demonstrate complementary metric-specific patterns of chronic stroke-related microstructural differences, characterized by lower MAP-MRI indices and FA and higher MD in the Stroke group.

### 3.6 Associations between regional diffusion metrics and clinical function

Clinical associations were assessed in participants with stroke with available clinical data (*n* = 15; Supplemental Figs. S2–S6). Across regional analyses, diffusion–function relationships were generally modest to moderate in magnitude, with the most consistent directional patterns observed for gait-related outcomes. Because these exploratory analyses evaluated five diffusion metrics across 13 anatomical regions, statistical significance was assessed using BH-FDR correction across all 65 ROI × metric comparisons separately within each clinical outcome.

For FGA, MAP-MRI measures generally showed positive correlations with functional performance across motor and subcortical regions, whereas MD generally showed inverse correlations (Fig. S2). Two nominal associations were observed within the ipsilesional thalamus: higher RTOP was associated with better FGA performance (Spearman ρ = 0.516, *p* = 0.049, q = 0.642), whereas higher MD was associated with poorer FGA performance (ρ = −0.580, *p* = 0.023, q = 0.642). These findings were directionally consistent with the broader regional correlation pattern but did not survive FDR correction.

For the 10-Meter Walk Test, no nominal associations were observed during comfortable walking (Fig. S3), although regional correlations generally trended toward negative associations for MAP-MRI measures and FA and positive associations for MD, consistent with lower completion times representing better performance. Fast walking demonstrated a similar but more pronounced directional pattern (Fig. S4), with two nominal associations within the ipsilesional thalamus. Higher FA was associated with faster walking, reflected by an inverse association with completion time (ρ = −0.632, *p* = 0.011, q = 0.546), whereas higher MD was associated with slower walking (ρ = 0.529, *p* = 0.043, q = 0.546). The ipsilesional thalamus showed the strongest FA and MD correlations with fast walking among the evaluated regions.

Fugl-Meyer associations were comparatively limited (Figs. S5–S6). Upper-extremity Fugl-Meyer scores showed no nominal associations with any regional diffusion measure, with correlations generally small to moderate in magnitude. For lower-extremity Fugl-Meyer scores, ipsilesional putamen FA demonstrated a nominal inverse association with performance (ρ = −0.590, *p* = 0.021, q = 0.938). Given its direction and lack of FDR-corrected significance, this association was interpreted cautiously.

Overall, nominal associations were observed for FGA, fast walking, and lower-extremity Fugl-Meyer outcomes, with the most consistent gait-related findings localized to the ipsilesional thalamus. None of the regional clinical associations survived BH-FDR correction across the 65 ROI × metric comparisons within any clinical outcome (Table S1). Accordingly, these findings are interpreted as exploratory patterns of diffusion–function correspondence rather than confirmatory clinical associations.

### 3.7 Retrospective power and lesion-location sensitivity analysis

Retrospective power estimates varied by diffusion metric, with median estimated power of 0.874 for RTOP, 0.857 for RTAP, 0.831 for RTPP, 0.702 for FA, and 0.973 for MD (Supplementary Tables S6–S8). Across the observed ROI-specific sample sizes, approximately 80% power at nominal α = 0.05 required an absolute rank-biserial effect size of approximately 0.50–0.52. Descriptive lesion-location-stratified results for cortical (*n* = 11), subcortical (*n* = 2), and multifocal (*n* = 5) infarcts are provided in Supplementary Table S9; no inferential subgroup comparisons were performed.

## 4 Discussion

### 4.1 Summary of key findings

The present study demonstrates complementary and region-dependent sensitivity of MAP-MRI and DTI to chronic stroke-related microstructural abnormalities. Using diffusion measures derived from a common multi-shell acquisition and harmonized preprocessing framework, substantial group differences were identified across corticospinal, subcortical, and cerebellar regions. MAP-derived RTOP, RTAP, and RTPP demonstrated widespread reductions in participants with stroke, with prominent effects within the ipsilesional CST and thalamus and additional abnormalities involving the caudate, putamen, and cerebellum. Among conventional DTI measures, FA demonstrated a more regionally restricted pattern, whereas MD was increased across all 13 evaluated ROIs and exhibited some of the largest group effects.

These regional findings remained robust after adjustment for age and sex. Significant group effects remained in 11 of 13 ROIs for RTOP, 10 of 13 for RTAP, and 9 of 13 for RTPP, compared with 6 of 13 for FA and all 13 for MD. Whole-brain voxel-wise analyses provided a complementary perspective: Whole-brain voxel-wise analyses provided a complementary perspective: following age and sex adjustment and stringent TFCE-FWE correction, significant abnormalities were identified across all MAP-MRI and DTI measures, with the spatial extent and distribution of corrected effects varying substantially by metric. MAP-MRI measures provided greater discrimination than FA in several motor and subcortical regions, whereas MD showed comparable or greater discrimination in others. Together, these findings indicate that MAP-MRI and DTI provide complementary information about chronic stroke-related tissue abnormalities, with their relative sensitivity depending on the diffusion metric, anatomical region, and spatial scale of analysis.

### 4.2 Subcortical and cerebellar DTI alterations in stroke

Prior DTI studies in stroke have predominantly characterized degeneration of major, highly coherent white matter pathways, most consistently the corticospinal tract, where tensor-derived metrics reliably capture Wallerian degeneration remote from the infarct (Werring et al., 2000; Puig et al., 2010). DTI abnormalities within subcortical structures, including the thalamus, basal ganglia, and cerebellum, have been reported less consistently. Increased MD within the ipsilesional thalamus following middle cerebral artery stroke has been described even when the thalamus is structurally spared (Buffon et al., 2005; Hervé et al., 2005), and targeted analyses have confirmed secondary degeneration within specific thalamic nuclei and their cortical projections during subacute and chronic stroke (Keser et al., 2021).

The present results are broadly consistent with this literature but demonstrate that DTI abnormalities are not limited to highly coherent white-matter pathways. MD was significantly increased in stroke across all evaluated ROIs, with particularly large effects with the ipsilesional CST and thalamus. FA showed a more selective pattern, with significant abnormalities in eight of 13 regions in the primary analysis and only six regions after adjustment for age and sex. This divergence between FA and MD likely reflect their sensitivity to different characteristics of tissue microstructure: FA primarily describes diffusion anisotropy, whereas MD reflects the overall magnitude of diffusion and may therefore remain sensitive to tissue abnormalities when directional organization is relatively preserved.

DTI abnormalities were also evident within the cerebellum and basal ganglia. Prior cerebellar studies have emphasized projection systems, crossed cerebellar diaschisis and corticocerebellar disconnection (Kim et al., 2005; Schulz et al., 2017; Yoo et al., 2023). In the present study, MD abnormalities were observed in both cerebellar gray and white matter, including crossed and non-crossed regions. Similarly, significant MD differences were identified within ipsilesional and contralesional striatal structures. These findings demonstrate that conventional DTI, particularly MD, remains highly sensitive to distributed chronic stroke-related tissue abnormalities and provide an important reference against which complementary information provided by MAP-MRI can be considered.

### 4.3 Complementary sensitivity of MAP-MRI metrics

MAP-MRI provides a conceptually different characterization of diffusion compared to conventional tensor modeling. Whereas DTI represents diffusion using a second-order tensor, MAP-MRI estimates the diffusion propagator without assuming a specific compartmental structure, permitting characterization of non-Gaussian diffusion across heterogenous gray-and white-matter environments (Özarslan et al., 2013). This distinction may be particularly relevant in regions with complex cytoarchitecture or heterogenous fiber orientation, where diffusion properties are not dominated by a single coherent direction (Tournier et al., 2011; Jones et al., 2013; O'Donnell and Westin, 2011).

In the present cohort, RTOP, RTAP, and RTPP demonstrated large group differences across corticospinal, thalamic, striatal, and cerebellar regions. Particularly strong lesion-relative effects were observed within the ipsilesional CST and thalamus, while significant abnormalities were also identified within contralesional subcortical structures and bilateral cerebellar regions. Regional effects remained robust after adjustment for age and sex, with significant differences retained in 11 of 13 ROIs for RTOP, 10 of 13 for RTAP, and 9 of 13 for RTPP. In comparison, significant adjusted effects were retained in 6 of 13 ROIs for FA and all 13 ROIs for MD. Whole-brain voxel-wise analysis similarly demonstrated significant TFCE-FWE–corrected abnormalities across all five-diffusion metrics, although their spatial distributions differed substantially by metric. RTOP and RTAP showed the most spatially extensive Control > Stroke effects, followed by RTPP and FA, whereas MD demonstrated extensive effects in the opposite, Stroke > Control direction. Together, the regional and whole-brain findings indicate that the relative sensitivity of MAP-MRI and DTI depends on the specific diffusion metric, anatomical region, and spatial scale of analysis.

Interpretation of these sensitivity patterns also requires consideration of differences in the diffusion information available to each model. Although DTI and MAP-MRI were derived from the same preprocessed diffusion acquisition, the amount and distribution of data used for model estimation differed. DTI was fitted using the b = 0 and b = 1000 s/mm^2^ measurements, including 16 non-collinear diffusion-encoding directions at b = 1000 s/mm^2^, whereas MAP-MRI incorporated the complete six-shell acquisition comprising 95 diffusion-weighted measurements. MAP-MRI therefore benefited from broader radial sampling and a greater number of diffusion measurements, which may contribute to greater estimation precision and statistical sensitivity independent of differences in model formulation. Conversely, although 16 non-collinear directions provide an overdetermined tensor fit, this represents more limited angular sampling than protocols optimized for high-precision tensor estimation, and previous simulation work has demonstrated decreasing uncertainty in tensor-derived measures with increasing diffusion-direction sampling (Jones, 2004). Accordingly, differences in the extent of significant findings between MAP-MRI and DTI likely reflect the combined effects of model formulation, b-value coverage, angular sampling, and estimation precision.

These findings should not, however, be interpreted as demonstrating global superiority of MAP-MRI over DTI. MD demonstrated significant abnormalities across all 13 ROIs in the primary analysis and retained all ROIs after demographic adjustment. Rather, the results suggest metric-specific sensitivity to different features of stroke-related tissue abnormality. RTOP reflects the probability of zero net displacement and is sensitive to restrictions on overall diffusion, whereas RTAP and RTPP characterize displacement perpendicular and parallel to the principal diffusion direction, respectively. Their regional patterns therefore provide information that is related to, but not equivalent to, FA and MD. The convergence and divergence of these measures across anatomical regions indicate that MAP-MRI and DTI capture related but non-equivalent aspects of diffusion behavior after stroke, supporting further evaluation of MAP-MRI as an additional diffusion-modeling approach.

### 4.4 Spatial distribution and network-level implications

The lesion-relative organization of the present analysis provides additional anatomical context for interpreting distributed diffusion abnormalities. Particularly large effects within the ipsilesional CST and thalamus are consistent with established patterns of tissue alteration associated with disruption of descending motor and corticothalamic pathways following supratentorial stroke. At the same time, significant abnormalities within contralesional subcortical structures and crossed and non-crossed cerebellar regions demonstrate that chronic stroke is associated with diffusion differences extending beyond the immediate lesion hemisphere. These spatial patterns are consistent with prior reports of post-stroke abnormalities associated with diaschises and secondary degeneration (Baudat et al., 2020; Weishaupt et al., 2015; Jiang et al., 2020; Sobesky et al., 2005; Yamauchi et al., 2022). Prior work from our laboratory has similarly demonstrated altered indirect structural connectivity in ipsilesional subcortical regions and bilateral cerebellum after stroke (Sotelo et al., 2020), while cerebellar-prefrontal structurofunctional connectivity has been associated with motor impairment, underscoring the role of cerebellar and subcortical hubs in large-scale network reorganization (Kalinosky et al., 2017; Kalinosky et al., 2013). The whole-brain analysis further demonstrated that diffusion abnormalities extended beyond the predefined ROIs, although their corrected spatial extent and distribution differed substantially across metrics. Following demographic adjustment and TFCE-FWE correction, RTOP and RTAP demonstrated spatially extensive Control > Stroke effects, RTPP showed a comparatively more restricted but still distributed pattern, and FA demonstrated the most spatially restricted Control > Stroke effects. MD showed extensive abnormalities in the opposite, Stroke > Control direction. Thus, evidence for distributed post-stroke diffusion changes was supported by both regional and whole-brain analyses, while the spatial pattern and direction of these differences depended on the diffusion measure examined.

Nevertheless, the present cross-sectional diffusion measurements do not directly establish that the observed remote abnormalities represent secondary degeneration, diaschisis, or network-level reorganization. Direct testing of these mechanisms would require longitudinal measurements and explicit characterization of the structural pathways connecting the lesion with remote regions. Accordingly, the present findings are most appropriately interpreted as demonstrated spatially distributed diffusion differences whose anatomical pattern is consistent with previously described remote post-stroke alterations. Future integration with tractography and structural connectivity analysis may determine whether these abnormalities preferentially occur within networks structurally connected to the infarct.

### 4.5 Clinical and translational implications

Conventional diffusion imaging biomarkers in stroke have largely focused on corticospinal tract integrity, commonly quantified using fractional anisotropy or lesion load within descending motor pathways (Puig et al. 2017; Moura et al. 2019). The present findings demonstrate that substantial diffusion abnormalities are also present within the thalamus, basal ganglia, and cerebellum. These structures participate in distributed motor and cognitive circuits, and the differing regional patterns observed across MAP-MRI and DTI suggest that the two approaches characterize complementary features of chronic stroke-related tissue abnormality.

The exploratory clinical analyses identified several nominally significant associations between regional diffusion measures and motor function. Nominal associations with FGA and fast 10-m walk performance were primarily localized to the ipsilesional thalamus, with an additional nominal association between ipsilesional putamen FA and lower-extremity Fugl-Meyer performance. None of these associations survived correction for multiple comparisons. Thus, although the observed patterns may help generate hypotheses regarding the functional relevance of regional diffusion abnormalities, the present data do not statistically establish clinical associations. Given the small clinical sample (*N* = 15), cross-sectional design, and multiple statistical comparisons, these analyses should be considered exploratory.

One potential extension of these findings is the application of advance diffusion measures to structural connectivity. Structural connectomes are typically constructed by parcellating the brain into regions and estimating white matter connections between them using diffusion MRI, with connections represented as weighted edges derived from measures such as streamline count, fractional anisotropy, or related indices (Hagmann et al., 2008; Rubinov & Sporns, 2010). Because structural connectivity estimates depend on the tractography approach, diffusion model, and definition of edge weight, their biological interpretation is influenced by how the underlying diffusion signal is represented (Cofts & Higham, 2011; Gast et al., 2024). Conventional tensor derived measures provide established markers of white matter integrity and remain particularly informative within coherent pathways such as the corticospinal tract. In the present study, however, MAP-MRI also demonstrated prominent abnormalities within subcortical and cerebellar regions, including the caudate, putamen, and cerebellar gray matter, where tissue architecture may not be adequately characterized by directional tensor measures alone. These observations raise the possibility that MAP-derived measures could provide complementary information for characterizing the integrity of distributed motor networks. Propagator-based measures may offer additional characterization of tissue within cortico-thalamo-striatal and cerebello-thalamo-cortical systems, potentially complementing conventional DTI-derived measures when constructing or weighting structural connectomes. Previous studies have demonstrated relationships between diffusion MRI-derived structural connectivity and motor or functional outcomes after stroke (Kalinosky et al., 2013, 2017; Schlemm et al., 2020), providing a rationale for evaluating whether MAP-informed connectivity measures capture additional clinically relevant information. Importantly, the present study does not establish that MAP-MRI produces more accurate structural connectomes or provides predictive information beyond conventional DTI. Rather, the present findings identify regional microstructural differences that motivate direct investigation of MAP-informed connectivity in stroke.

### 4.6 Limitations

Several limitations should be considered when interpreting these findings. First, the modest sample size and heterogeneous stroke cohort may limit generalizability across lesion locations and clinical presentations. Retrospective sensitivity analyses indicated that the available sample was primarily capable of detecting relatively large group differences, with approximately 80% power corresponding to an absolute rank biserial effect $r_{\mathrm{r\beta}}$ ≈ 0.50–0.52 at nominal α = 0.05. Accordingly, nonsignificant findings, particularly for smaller observed effects, should not be interpreted as evidence of equivalence. The sample size also limited the ability to reliably evaluate differences related to specific lesion territories. Lesion-location-stratified summaries were therefore exploratory and descriptive only because of the small and unequal subgroup sizes, and no inferential comparisons were performed between cortical, subcortical, and multifocal lesion groups. Although the lesion-relative framework improved anatomical consistency across participants, the cross-sectional design cannot determine whether abnormalities outside the primary lesion reflect secondary degeneration, pre-existing differences, or other post-stroke processes. Accordingly, distributed abnormalities are interpreted as remote microstructural differences rather than direct evidence of specific degenerative mechanisms. Second, the clinical analyses were limited to participants with available outcome data and should be considered exploratory, particularly because associations did not remain significant under the more conservative correction across all ROI × metric comparisons. Third, the present study evaluated regional and whole-brain diffusion changes rather than structural connectivity directly. Whether MAP-derived abnormalities correspond to alterations within specific cortico-thalamo-striatal or cerebello-thalamo-cortical pathways remains to be established. Finally, although age and sex were included as covariates in sensitivity analyses, the modest sample size and residual between-group demographic differences mean that confounding by demographic factors cannot be completely excluded.

### 4.7 Future directions and conclusions

Future studies should extend these findings in larger longitudinal cohorts to determine how MAP-MRI abnormalities evolve after stroke and whether they provide clinically relevant information beyond conventional DTI. Of particular interest is whether MAP-derived measures can complement DTI in characterizing residual tissue integrity within distributed motor systems and improve associations with functional outcomes. Incorporating MAP-derived microstructural information into tractography and structural connectivity analyses may further determine whether the regional abnormalities identified here correspond to disruption of cortico-thalamo-striatal and cerbello-thalamo-cortical circuitry. Reproducibility across scanners and acquisition protocols and incremental value of MAP-derived measures over established measures should also be evaluated before their application as predictive biomarkers.

In conclusion, using a harmonized diffusion acquisition and preprocessing framework, the present study demonstrates spatially distributed diffusion differences in chronic stroke across both MAP-MRI and DTI measures. MAP-derived metrics identified regional abnormalities across major motor pathways, subcortical structures, and the cerebellum, and whole-brain analyses demonstrated metric-dependent differences in the spatial extent of corrected abnormalities. DTI provided complementary information, with MD demonstrating particularly widespread abnormalities and FA showing strong effects within selected motor-related regions. Together, these findings demonstrate that MAP-MRI and DTI capture overlapping but distinct features of chronic-stroke related tissue alteration and support further evaluation of advanced multi-shell diffusion modeling as an additional approach for characterizing distributed structural consequences of stroke.

The following are the supplementary data related to this article.

**Supplementary Fig. S1:**
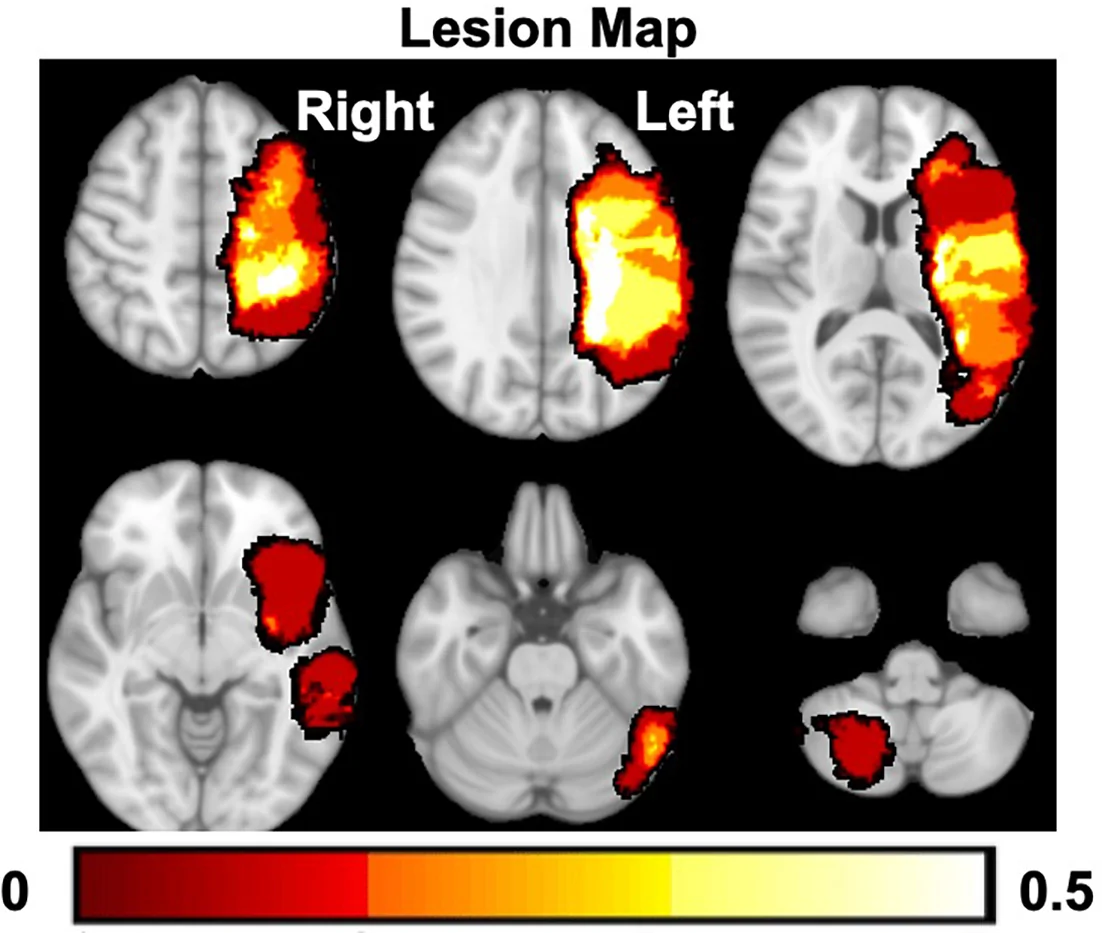
Lesion distribution within the stroke cohort. Individual lesion masks were transformed to MNI space and oriented according to lesion-relative anatomy for group analysis. Cerebral lesions were standardized to the left (ipsilesional) hemisphere, while cerebellar lesions were retained in the right (contralesional/crossed) hemisphere to preserve their anatomical relationship to the cerebral lesion side. The color scale represents the proportion of participants with lesion involvement at each voxel (0–0.5), with brighter intensities indicating greater lesion overlap.

**Supplementary Fig. S2:**
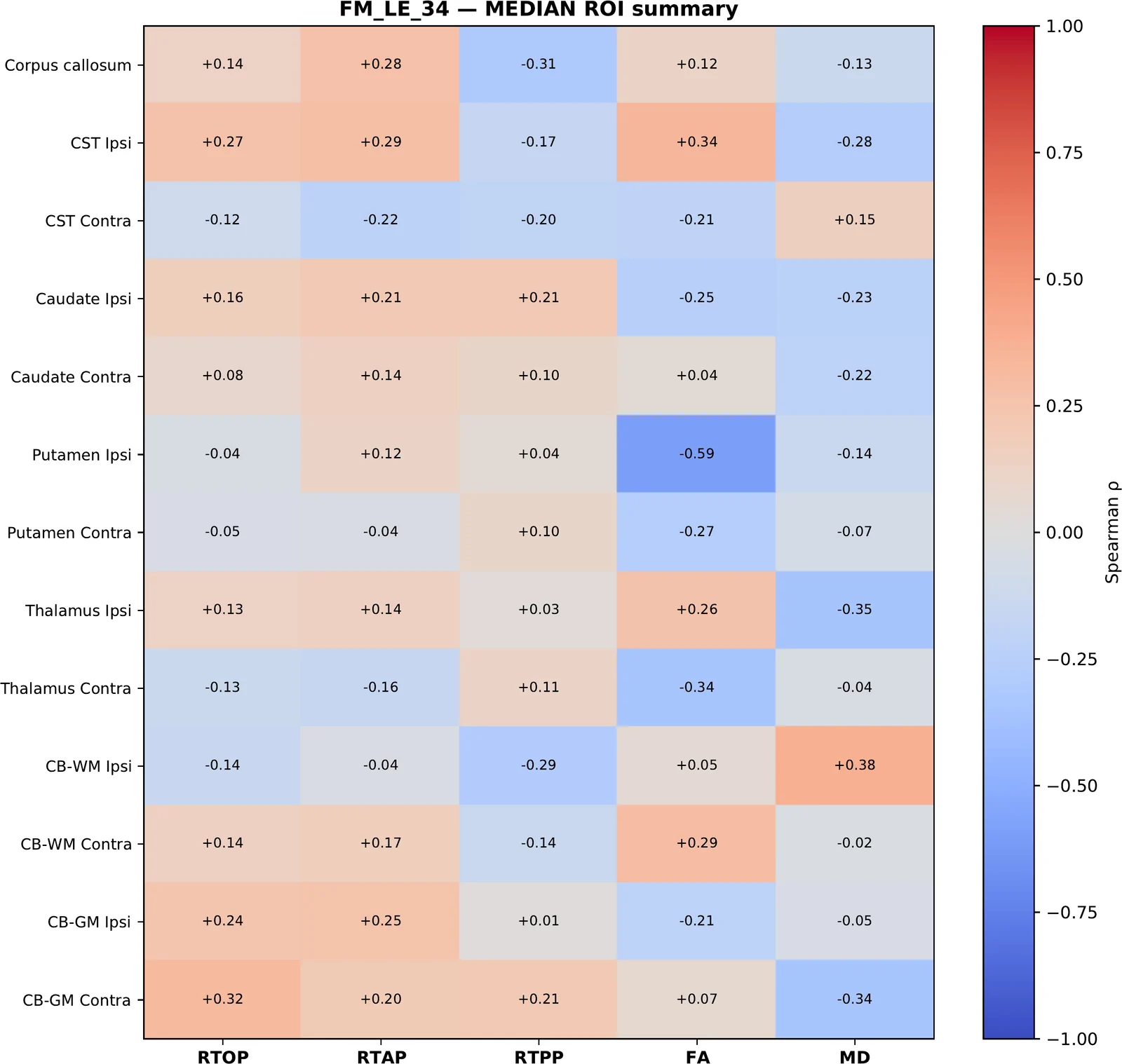
The heatmap displays Spearman correlation coefficient (ρ) between subject-level median regional diffusion measures and Functional Gait Assessment (FGA) scores in participants with chronic stroke (n=15). Correlations are shown for RTOP, RTAP, RTPP, FA, and MD across 13 lesion-relative regions of interest. Positive correlations indicate that higher diffusion metric values are associated better FGA performance, whereas negative correlations indicate inverse associations. Statistical significance was evaluated using BH-FDR correction across all 65 ROI × metric comparisons within the clinical outcome.

**Supplementary Fig. S3:**
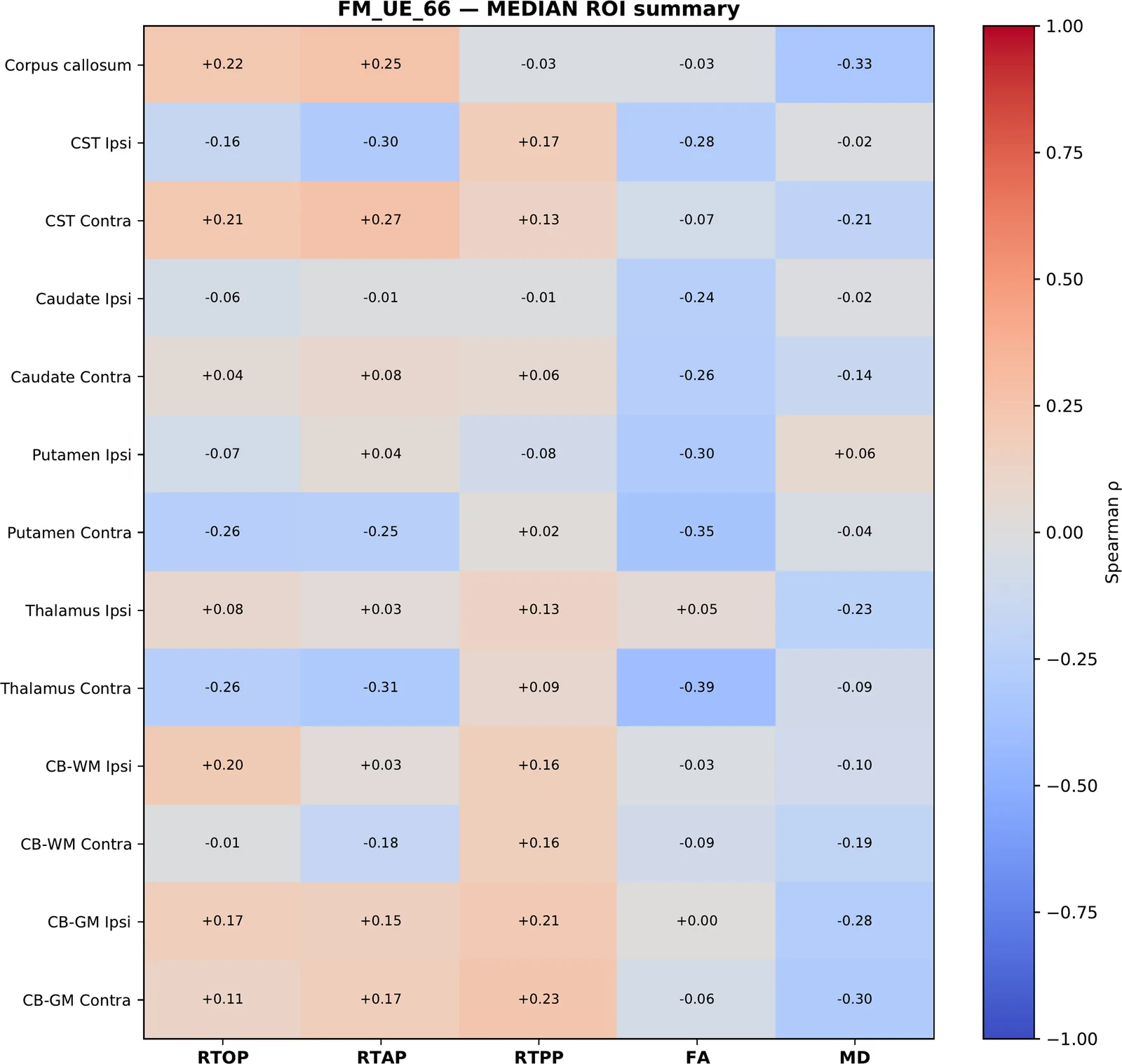
The heatmap displays Spearman correlation coefficient (ρ) between subject-level median regional diffusion measures and comfortable 10-meter walk (comfortable walking speed) test scores in participants with chronic stroke (n=15). Correlations are shown for RTOP, RTAP, RTPP, FA, and MD across 13 lesion-relative regions of interest. Because lower completion time indicates better walking performance, negative correlations indicate that higher diffusion metric values are associated with faster walking, whereas positive correlations indicate associations with slower walking. Statistical significance was evaluated using BH-FDR correction across all 65 ROI × metric comparisons within the clinical outcome.

**Supplementary Fig. S4:**
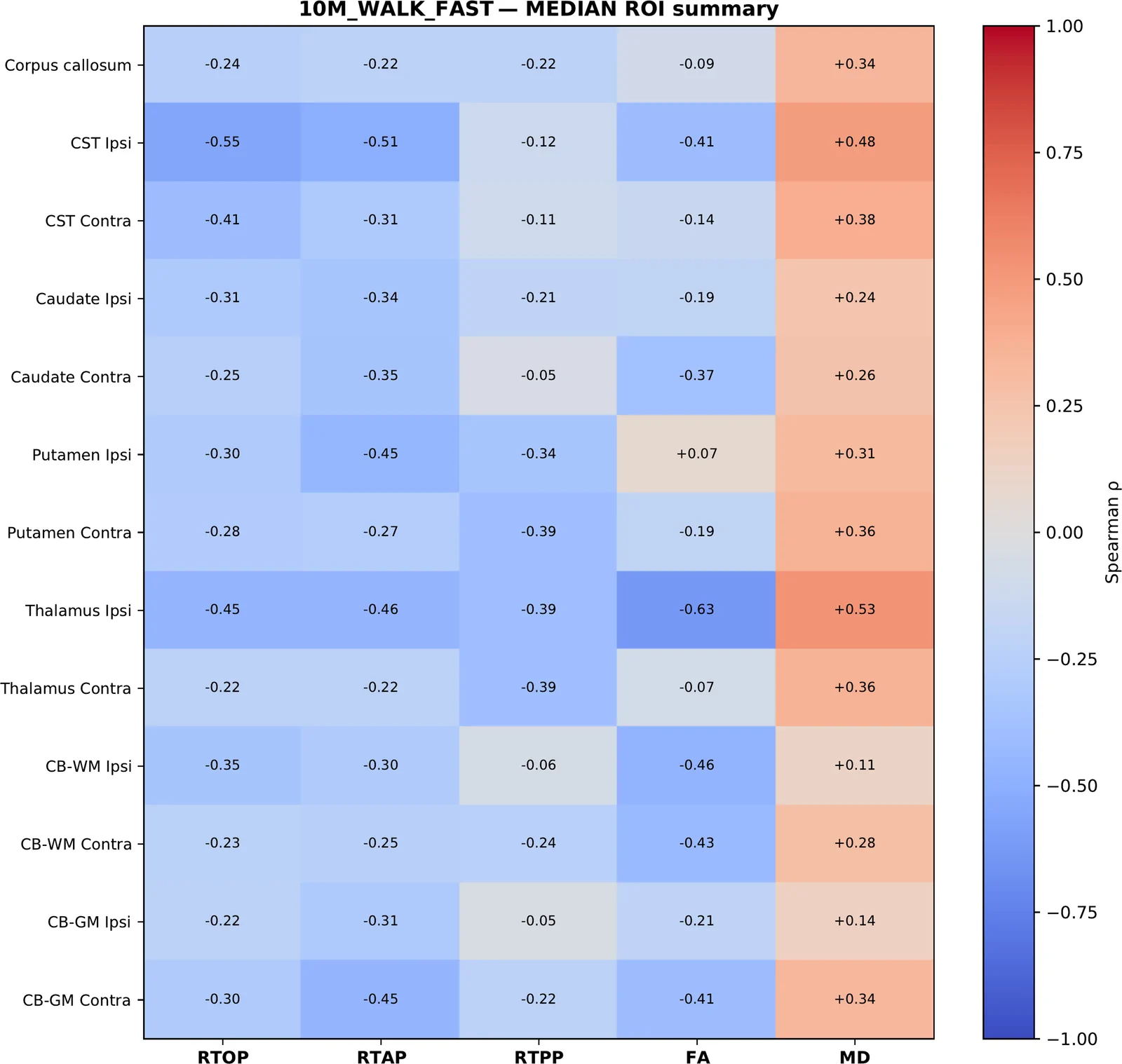
The heatmap displays Spearman correlation coefficient (ρ) between subject-level median regional diffusion measures and comfortable 10-meter walk (fast) test scores in participants with chronic stroke (n=15). Correlations are shown for RTOP, RTAP, RTPP, FA, and MD across 13 lesion-relative regions of interest. Because lower completion time indicates better walking performance, negative correlations indicate that higher diffusion metric values are associated with faster walking, whereas positive correlations indicate associations with slower walking. Statistical significance was evaluated using BH-FDR correction across all 65 ROI × metric comparisons within the clinical outcome.

**Supplementary Fig. S5:**
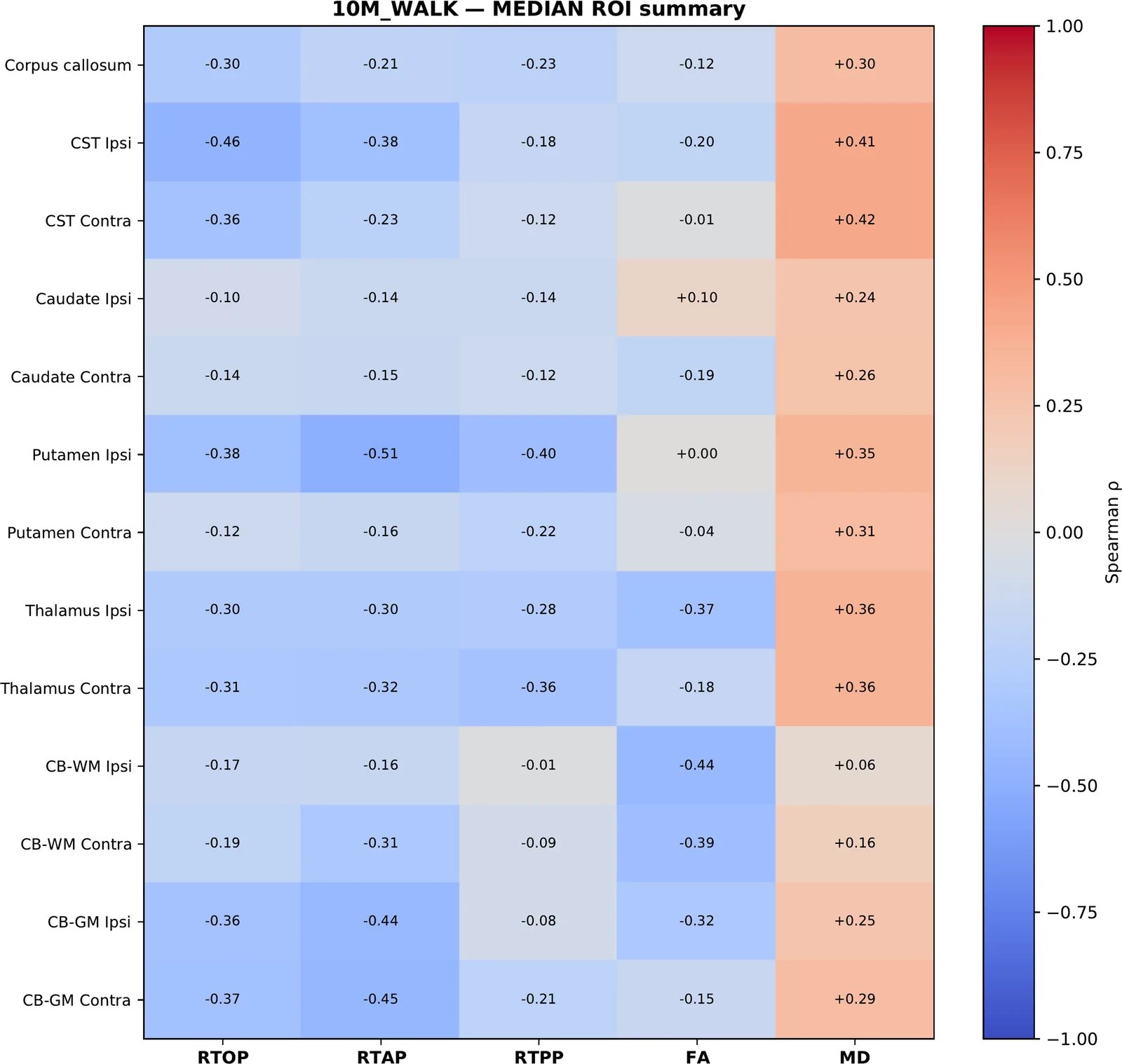
The heatmap displays Spearman correlation coefficient (ρ) between subject-level median regional diffusion measures and comfortable 10-meter walk test scores in participants with chronic stroke (n=15). Correlations are shown for RTOP, RTAP, RTPP, FA, and MD across 13 lesion-relative regions of interest. Because lower completion time indicates better walking performance, negative correlations indicate that higher diffusion metric values are associated with faster walking, whereas positive correlations indicate associations with slower walking. Statistical significance was evaluated using BH-FDR correction across all 65 ROI × metric comparisons within the clinical outcome.

Supplementary material 6 Correlations between regional diffusion measures and clinical outcomes in participants with chronic stroke.

Supplementary material 7 Clinical Assessment table, displaying time between MRI scan and Clinical Assessments.

Supplementary material 8 Leave-one-out influence analysis of significant regional diffusion findings. For each of the 54 ROI × metric comparisons significant in the finalized full-cohort analysis (BH-FDR q<0.05 across 65 comparisons), each stroke participant was omitted individually and the Control-versus-Stroke rank-biserial correlation (rᵣᵇ) was recalculated. LOO range gives the minimum–maximum rᵣᵇ across the 18 leave-one-out iterations. Max |Δrᵣᵇ| is the largest absolute deviation from the full-cohort effect. q<0.05 LOO gives the number of 18 iterations that remained significant after separately reapplying BH-FDR across all 65 tests. Directional stability is the primary influence criterion.

Supplementary material 9 Participant-level lesion characteristics and available clinical severity measures.

Supplementary material 10 Direct lesion-overlap sensitivity analysis.

Supplementary material 11 Retrospective Mann–Whitney U power summary for primary median-based Control-versus-Stroke ROI comparisons.

Supplementary material 12 Minimum detectable effect at 80% power across observed ROI sample Size.

Supplementary material 13 Comparison-level retrospective Mann–Whitney U power estimates for primary median-based Control-versus-Stroke ROI comparisons.

Supplementary material 14 Descriptive diffusion measures stratified by lesion location.

## Statement on Use of AI

During the preparation of this work, the authors used ChatGPT to assist in text editing and to draft keywords and highlights for the manuscript. Google ScholarLabs was used to identify potentially missed literature. The author(s) reviewed and edited the output as needed and take full responsibility for the content of the published article.

## CRediT authorship contribution statement

**Ricardo J. Vega:** Writing – review & editing, Writing – original draft, Visualization, Validation, Software, Methodology, Investigation, Formal analysis, Data curation. **Wesely T. Richerson:** Writing – review & editing, Methodology, Formal analysis, Conceptualization. **Max O. Krucoff:** Writing – review & editing, Visualization, Supervision, Formal analysis. **Brian D. Schmit:** Writing – review & editing, Supervision, Resources, Project administration, Methodology, Funding acquisition, Conceptualization.

## Ethics declaration

**Informed consent and patient details**: Written informed consent to take part in the study and to publish the article has been obtained from all participants or their legal representatives. The privacy rights of participants have been observed.

**Studies in Human**: This study was performed in compliance with relevant laws, regulatory frameworks and guidelines where the research took place. This study was approved by the Medical College of Wisconsin Institutional Review Board. (Approval No. PRO00046740).

## Funding

This work was supported by the Hammes Family Endowment at Marquette University and the Medical College of Wisconsin.

## Declaration of competing interest

This study was funded by the Hammes Family Endowment to the Joint Department of Biomedical Engineering at Marquette University and the Medica College of Wisconsin. The authors declare that they have no known competing financial interests or personal relationships that could have appeared to influence the work reported in this paper.

## Data Availability

Data will be made available on request.
