# Supplementary material for: Characterizing post-stroke changes in brain microstructure using the mean apparent propagator model
Source: Neuroimage Clin. 2026 Oct 21;52:104076. doi: 10.1016/j.nicl.2026.104076 (PMC13722559; doi:10.1016/j.nicl.2026.104076)
Supplement: Supplementary file 1 [file mmc10.docx]

**Supplementary Table S5. Direct lesion-overlap sensitivity analysis.**

| **ROI** | **Valid-mask N** | **Direct overlap N** | **Non-overlap N** | **RTOP** | **RTAP** | **RTPP** | **FA** | **MD** |
| --- | --- | --- | --- | --- | --- | --- | --- | --- |
| Corpus callosum | 12 | 3 | 9 | 0.640/.012 -> 0.520/.061 | 0.728/.005 -> **0.637/.027** | -0.456/.057 -> -0.520/.061 | 0.746/.005 -> **0.661/.027** | -0.693/.007 -> **-0.602/.034** |
| CST Ipsi | 11 | 4 | 7 | 0.799/.004 -> **0.699/.027** | 0.665/.011 -> 0.474/.108 | 0.675/.011 -> **0.880/.020** | 0.464/.059 -> 0.158/.590 | -0.866/.004 -> **-0.789/.027** |
| CST Contra | 12 | 2 | 10 | 0.526/.035 -> 0.463/.081 | 0.456/.057 -> 0.442/.092 | 0.482/.046 -> 0.379/.136 | 0.088/.711 -> 0.084/.731 | -0.553/.028 -> -0.463/.081 |
| Caudate Ipsi | 13 | 3 | 10 | 0.644/.011 -> 0.537/.053 | 0.725/.005 -> **0.642/.027** | 0.547/.028 -> 0.495/.061 | -0.053/.818 -> -0.126/.615 | -0.749/.004 -> **-0.674/.027** |
| Caudate Contra | 13 | 0 | 13 | 0.457/.051 -> 0.457/.061 | 0.474/.046 -> 0.474/.056 | 0.377/.101 -> 0.377/.112 | -0.150/.505 -> -0.150/.522 | -0.676/.007 -> **-0.676/.024** |
| Putamen Ipsi | 13 | 4 | 9 | 0.514/.035 -> 0.392/.136 | 0.409/.076 -> 0.392/.136 | 0.757/.004 -> **0.649/.027** | -0.239/.288 -> -0.205/.437 | -0.757/.004 -> **-0.649/.027** |
| Putamen Contra | 13 | 2 | 11 | 0.287/.208 -> 0.273/.265 | 0.279/.219 -> 0.244/.322 | 0.474/.046 -> 0.426/.092 | -0.215/.339 -> -0.292/.237 | -0.692/.007 -> **-0.636/.027** |
| Thalamus Ipsi | 13 | 2 | 11 | 0.255/.258 -> 0.120/.615 | 0.320/.159 -> 0.196/.429 | 0.579/.020 -> 0.502/.056 | 0.498/.040 -> 0.407/.108 | -0.781/.004 -> **-0.742/.020** |
| Thalamus Contra | 13 | 0 | 13 | 0.344/.134 -> 0.344/.136 | 0.320/.159 -> 0.320/.165 | 0.409/.076 -> 0.409/.092 | 0.344/.134 -> 0.344/.136 | -0.474/.046 -> -0.474/.056 |
| CB-WM Ipsi | 13 | 0 | 13 | 0.474/.046 -> 0.474/.056 | 0.611/.014 -> **0.611/.027** | 0.263/.246 -> 0.263/.259 | 0.514/.035 -> **0.514/.042** | -0.336/.142 -> -0.336/.145 |
| CB-WM Contra | 13 | 0 | 13 | 0.603/.015 -> **0.603/.027** | 0.741/.004 -> **0.741/.020** | 0.190/.396 -> 0.190/.423 | 0.636/.011 -> **0.636/.027** | -0.538/.028 -> **-0.538/.033** |
| CB-GM Ipsi | 13 | 3 | 10 | 0.506/.038 -> **0.632/.027** | 0.409/.076 -> 0.495/.061 | 0.457/.051 -> **0.621/.027** | 0.401/.081 -> 0.389/.133 | -0.490/.043 -> **-0.642/.027** |
| CB-GM Contra | 13 | 0 | 13 | 0.538/.028 -> **0.538/.033** | 0.385/.095 -> 0.385/.108 | 0.482/.046 -> 0.482/.056 | 0.417/.074 -> 0.417/.086 | -0.555/.027 -> **-0.555/.030** |

**Note.** Each metric cell is formatted as valid-mask baseline ($r_{r\beta}$) -> non-overlap ($r_{r\beta}$). The non-overlap analysis excludes stroke participants whose quantitative lesion mask directly overlapped that ROI. Bold non-overlap values indicate q < 0.05. BH-FDR correction was applied across all 65 ROI x diffusion-metric comparisons within the non-overlap sensitivity family. The valid-mask baseline is the appropriate comparator because participants without a reliable quantitative lesion mask were assigned unknown overlap status and were not classified as non-overlapping. The direct-overlap criterion was at least one overlapping voxel. Positive r_rb indicates Control > Stroke; negative r_rb indicates Stroke > Control.

**Retained significance after direct-overlap exclusion:** RTOP 4/13, RTAP 4/13, RTPP 3/13, FA 3/13, and MD 10/13.
