# Supplementary material for: Characterizing post-stroke changes in brain microstructure using the mean apparent propagator model
Source: Neuroimage Clin. 2026 Oct 21;52:104076. doi: 10.1016/j.nicl.2026.104076 (PMC13722559; doi:10.1016/j.nicl.2026.104076)
Supplement: Supplementary file 2 [file mmc11.docx]

Table S6. Retrospective Mann–Whitney U power summary for primary median-based Control-versus-Stroke ROI comparisons.

| **Metric** | **FDR-significant ROI tests** | **Median \|rᵣᵦ\|** | **Median MWU power** | **Power range** | **ROI tests with power ≥ 0.80** | **Median power: FDR significant** | **Median power: FDR nonsignificant** |
| --- | --- | --- | --- | --- | --- | --- | --- |
| RTOP | 11/13 | 0.561 | 0.874 | 0.454–1.000 | 10/13 | 0.891 | 0.459 |
| RTAP | 12/13 | 0.566 | 0.857 | 0.465–0.999 | 8/13 | 0.868 | 0.465 |
| RTPP | 10/13 | 0.536 | 0.831 | 0.349–0.999 | 7/13 | 0.893 | 0.468 |
| FA | 8/13 | 0.467 | 0.702 | 0.050–0.998 | 4/13 | 0.843 | 0.100 |
| MD | 13/13 | 0.678 | 0.973 | 0.775–1.000 | 12/13 | 0.973 | — |

**Abbreviations:** MWU, Mann–Whitney U; rᵣᵦ, rank-biserial correlation; FDR, false discovery rate.

**Note:** Retrospective power estimates are provided for descriptive context and were not used for statistical inference. FDR refers to the BH-FDR-adjusted primary ROI results.
