# Supplementary material for: Characterizing post-stroke changes in brain microstructure using the mean apparent propagator model
Source: Neuroimage Clin. 2026 Oct 21;52:104076. doi: 10.1016/j.nicl.2026.104076 (PMC13722559; doi:10.1016/j.nicl.2026.104076)
Supplement: Supplementary file 3 [file mmc12.docx]

Table S7. Minimum detectable effect at 80% power across observed ROI sample Size.

| **Control n** | **Stroke n** | **MDE Cohen's d α = 0.05** | **MDE \|rᵣᵦ\| α = 0.05** | **MDE Cohen's d α = 0.05/13** | **MDE \|rᵣᵦ\| α = 0.05/13** |
| --- | --- | --- | --- | --- | --- |
| 20 | 15 | 0.998 | 0.520 | 1.380 | 0.671 |
| 20 | 16 | 0.979 | 0.511 | 1.353 | 0.661 |
| 20 | 17 | 0.963 | 0.504 | 1.328 | 0.652 |
| 20 | 18 | 0.948 | 0.497 | 1.306 | 0.644 |

**MDE = minimum detectable effect at 80% power.** Across the observed ROI-specific sample sizes, 80% power at nominal α = 0.05 required approximately |rᵣᵦ| = 0.50–0.52 (Cohen's d ≈ 0.95–1.00).

**Note:** Retrospective power estimates are provided for descriptive context and were not used for statistical inference. Results calculated at α = 0.05/13 are included only as a conservative multiplicity benchmark and should not be interpreted as BH-FDR-adjusted power.
