# Supplementary material for: Characterizing post-stroke changes in brain microstructure using the mean apparent propagator model
Source: Neuroimage Clin. 2026 Oct 21;52:104076. doi: 10.1016/j.nicl.2026.104076 (PMC13722559; doi:10.1016/j.nicl.2026.104076)
Supplement: Supplementary file 4 [file mmc13.docx]

Table S8. Comparison-level retrospective Mann–Whitney U power estimates for primary median-based Control-versus-Stroke ROI comparisons.

| **Metric** | **ROI** | **Control n** | **Stroke n** | **Rank-biserial r** | **\|r\|** | **BH-FDR q** | **FDR sig.** | **MWU power α=.05** | **Power ≥.80** | **MWU power α=.05/13** |
| --- | --- | --- | --- | --- | --- | --- | --- | --- | --- | --- |
| **RTOP** | **Corpus callosum** | **20** | **17** | **0.715** | **0.715** | **0.001** | **Yes** | **0.986** | **Yes** | **0.868** |
| RTOP | CST Ipsi | 20 | 15 | 0.846 | 0.846 | <0.001 | Yes | 1.000 | Yes | 0.989 |
| RTOP | CST Contra | 20 | 16 | 0.618 | 0.618 | 0.005 | Yes | 0.926 | Yes | 0.641 |
| RTOP | Caudate Ipsi | 20 | 17 | 0.703 | 0.703 | 0.001 | Yes | 0.984 | Yes | 0.853 |
| RTOP | Caudate Contra | 20 | 17 | 0.548 | 0.548 | 0.007 | Yes | 0.846 | Yes | 0.468 |
| RTOP | Putamen Ipsi | 20 | 17 | 0.560 | 0.560 | 0.007 | Yes | 0.874 | Yes | 0.504 |
| RTOP | Putamen Contra | 20 | 17 | 0.356 | 0.356 | 0.071 | No | 0.454 | No | 0.115 |
| RTOP | Thalamus Ipsi | 20 | 17 | 0.356 | 0.356 | 0.071 | No | 0.464 | No | 0.119 |
| RTOP | Thalamus Contra | 20 | 17 | 0.461 | 0.461 | 0.022 | Yes | 0.685 | No | 0.274 |
| RTOP | Cerebellar WM Ipsi | 20 | 18 | 0.544 | 0.544 | 0.007 | Yes | 0.852 | Yes | 0.492 |
| RTOP | Cerebellar WM Contra | 20 | 18 | 0.561 | 0.561 | 0.007 | Yes | 0.873 | Yes | 0.524 |
| RTOP | Cerebellar GM Ipsi | 20 | 18 | 0.579 | 0.579 | 0.006 | Yes | 0.891 | Yes | 0.581 |
| RTOP | Cerebellar GM Contra | 20 | 18 | 0.620 | 0.620 | 0.004 | Yes | 0.940 | Yes | 0.689 |
| **RTAP** | **Corpus callosum** | 20 | **17** | **0.765** | **0.765** | **<0.001** | **Yes** | **0.997** | **Yes** | **0.939** |
| RTAP | CST Ipsi | 20 | 15 | 0.740 | 0.740 | <0.001 | Yes | 0.992 | Yes | 0.871 |
| RTAP | CST Contra | 20 | 16 | 0.566 | 0.566 | 0.008 | Yes | 0.857 | Yes | 0.499 |
| RTAP | Caudate Ipsi | 20 | 17 | 0.777 | 0.777 | <0.001 | Yes | 0.999 | Yes | 0.956 |
| RTAP | Caudate Contra | 20 | 17 | 0.567 | 0.567 | 0.008 | Yes | 0.879 | Yes | 0.520 |
| RTAP | Putamen Ipsi | 20 | 17 | 0.449 | 0.449 | 0.027 | Yes | 0.650 | No | 0.246 |
| RTAP | Putamen Contra | 20 | 17 | 0.356 | 0.356 | 0.071 | No | 0.465 | No | 0.114 |
| RTAP | Thalamus Ipsi | 20 | 17 | 0.418 | 0.418 | 0.037 | Yes | 0.601 | No | 0.195 |
| RTAP | Thalamus Contra | 20 | 17 | 0.455 | 0.455 | 0.027 | Yes | 0.677 | No | 0.262 |
| RTAP | Cerebellar WM Ipsi | 20 | 18 | 0.632 | 0.632 | 0.003 | Yes | 0.950 | Yes | 0.723 |
| RTAP | Cerebellar WM Contra | 20 | 18 | 0.708 | 0.708 | <0.001 | Yes | 0.986 | Yes | 0.875 |
| RTAP | Cerebellar GM Ipsi | 20 | 18 | 0.509 | 0.509 | 0.012 | Yes | 0.795 | No | 0.409 |
| RTAP | Cerebellar GM Contra | 20 | 18 | 0.550 | 0.550 | 0.008 | Yes | 0.853 | Yes | 0.503 |
| **RTPP** | **Corpus callosum** | 20 | **17** | **-0.412** | **0.412** | **0.047** | **Yes** | **0.574** | **No** | **0.180** |
| RTPP | CST Ipsi | 20 | 15 | 0.761 | 0.761 | 0.001 | Yes | 0.994 | Yes | 0.915 |
| RTPP | CST Contra | 20 | 16 | 0.487 | 0.487 | 0.024 | Yes | 0.717 | No | 0.311 |
| RTPP | Caudate Ipsi | 20 | 17 | 0.567 | 0.567 | 0.009 | Yes | 0.881 | Yes | 0.519 |
| RTPP | Caudate Contra | 20 | 17 | 0.368 | 0.368 | 0.067 | No | 0.476 | No | 0.125 |
| RTPP | Putamen Ipsi | 20 | 17 | 0.783 | 0.783 | <0.001 | Yes | 0.999 | Yes | 0.962 |
| RTPP | Putamen Contra | 20 | 17 | 0.536 | 0.536 | 0.012 | Yes | 0.831 | Yes | 0.435 |
| RTPP | Thalamus Ipsi | 20 | 17 | 0.598 | 0.598 | 0.006 | Yes | 0.911 | Yes | 0.592 |
| RTPP | Thalamus Contra | 20 | 17 | 0.443 | 0.443 | 0.035 | Yes | 0.668 | No | 0.239 |
| RTPP | Cerebellar WM Ipsi | 20 | 18 | 0.363 | 0.363 | 0.067 | No | 0.468 | No | 0.131 |
| RTPP | Cerebellar WM Contra | 20 | 18 | 0.310 | 0.310 | 0.111 | No | 0.349 | No | 0.081 |
| RTPP | Cerebellar GM Ipsi | 20 | 18 | 0.585 | 0.585 | 0.006 | Yes | 0.911 | Yes | 0.609 |
| RTPP | Cerebellar GM Contra | 20 | 18 | 0.585 | 0.585 | 0.006 | Yes | 0.906 | Yes | 0.593 |
| **FA** | **Corpus callosum** | **20** | **17** | **0.783** | **0.783** | **<0.001** | **Yes** | **0.998** | **Yes** | **0.962** |
| FA | CST Ipsi | 20 | 15 | 0.516 | 0.516 | 0.026 | Yes | 0.770 | No | 0.341 |
| FA | CST Contra | 20 | 16 | 0.191 | 0.191 | 0.449 | No | 0.149 | No | 0.024 |
| FA | Caudate Ipsi | 20 | 17 | 0.009 | 0.009 | 0.975 | No | 0.050 | No | 0.003 |
| FA | Caudate Contra | 20 | 17 | 0.102 | 0.102 | 0.663 | No | 0.082 | No | 0.007 |
| FA | Putamen Ipsi | 20 | 17 | -0.207 | 0.207 | 0.427 | No | 0.175 | No | 0.024 |
| FA | Putamen Contra | 20 | 17 | -0.127 | 0.127 | 0.622 | No | 0.100 | No | 0.008 |
| FA | Thalamus Ipsi | 20 | 17 | 0.616 | 0.616 | 0.007 | Yes | 0.934 | Yes | 0.642 |
| FA | Thalamus Contra | 20 | 17 | 0.467 | 0.467 | 0.032 | Yes | 0.702 | No | 0.276 |
| FA | Cerebellar WM Ipsi | 20 | 18 | 0.602 | 0.602 | 0.007 | Yes | 0.926 | Yes | 0.637 |
| FA | Cerebellar WM Contra | 20 | 18 | 0.596 | 0.596 | 0.007 | Yes | 0.916 | Yes | 0.627 |
| FA | Cerebellar GM Ipsi | 20 | 18 | 0.433 | 0.433 | 0.041 | Yes | 0.638 | No | 0.242 |
| FA | Cerebellar GM Contra | 20 | 18 | 0.485 | 0.485 | 0.026 | Yes | 0.749 | No | 0.350 |
| **MD** | **Corpus callosum** | **20** | **17** | **-0.728** | **0.728** | **<0.001** | **Yes** | **0.994** | **Yes** | **0.896** |
| MD | CST Ipsi | 20 | 15 | -0.895 | 0.895 | <0.001 | Yes | 1.000 | Yes | 0.998 |
| MD | CST Contra | 20 | 16 | -0.625 | 0.625 | 0.002 | Yes | 0.938 | Yes | 0.654 |
| MD | Caudate Ipsi | 20 | 17 | -0.721 | 0.721 | <0.001 | Yes | 0.991 | Yes | 0.884 |
| MD | Caudate Contra | 20 | 17 | -0.678 | 0.678 | 0.001 | Yes | 0.973 | Yes | 0.794 |
| MD | Putamen Ipsi | 20 | 17 | -0.734 | 0.734 | <0.001 | Yes | 0.993 | Yes | 0.915 |
| MD | Putamen Contra | 20 | 17 | -0.678 | 0.678 | 0.001 | Yes | 0.978 | Yes | 0.795 |
| MD | Thalamus Ipsi | 20 | 17 | -0.827 | 0.827 | <0.001 | Yes | 0.999 | Yes | 0.987 |
| MD | Thalamus Contra | 20 | 17 | -0.591 | 0.591 | 0.003 | Yes | 0.909 | Yes | 0.574 |
| MD | Cerebellar WM Ipsi | 20 | 18 | -0.497 | 0.497 | 0.010 | Yes | 0.775 | No | 0.373 |
| MD | Cerebellar WM Contra | 20 | 18 | -0.608 | 0.608 | 0.002 | Yes | 0.933 | Yes | 0.650 |
| MD | Cerebellar GM Ipsi | 20 | 18 | -0.620 | 0.620 | 0.002 | Yes | 0.939 | Yes | 0.687 |
| MD | Cerebellar GM Contra | 20 | 18 | -0.626 | 0.626 | 0.002 | Yes | 0.947 | Yes | 0.702 |

**Note:** Retrospective power estimates are provided for descriptive context and were not used for statistical inference. Results calculated at α = 0.05/13 are included only as a conservative multiplicity benchmark and should not be interpreted as BH-FDR-adjusted power. FDR significance reflects the BH-FDR-adjusted primary ROI analysis.
