# Supplementary material for: Characterizing post-stroke changes in brain microstructure using the mean apparent propagator model
Source: Neuroimage Clin. 2026 Oct 21;52:104076. doi: 10.1016/j.nicl.2026.104076 (PMC13722559; doi:10.1016/j.nicl.2026.104076)
Supplement: Supplementary file 5 [file mmc14.docx]

**Table S9. Descriptive diffusion measures stratified by lesion location**

*Lesion-location subgroups: cortical n = 11; subcortical n = 2; multifocal n = 5.*

| **Metric** | **ROI** | **Cortical n** | **Cortical median [IQR]** | **Subcortical n** | **Subcortical median [IQR]** | **Multifocal n** | **Multifocal median [IQR]** |
| --- | --- | --- | --- | --- | --- | --- | --- |
| **RTOP** | Corpus callosum | 10 | 7.133e+05 [4.917e+05, 8.428e+05] | 2 | 7.814e+05 [6.949e+05, 8.680e+05] | 5 | 6.664e+05 [5.967e+05, 7.042e+05] |
| **RTOP** | CST Ipsi | 9 | 8.047e+05 [6.873e+05, 1.057e+06] | 2 | 1.008e+06 [9.675e+05, 1.048e+06] | 4 | 7.393e+05 [6.170e+05, 8.318e+05] |
| **RTOP** | CST Contra | 11 | 1.030e+06 [7.993e+05, 1.086e+06] | 1 | 1.022e+06 [—] | 4 | 8.287e+05 [6.736e+05, 9.546e+05] |
| **RTOP** | Caudate Ipsi | 11 | 3.110e+05 [2.200e+05, 3.735e+05] | 2 | 3.807e+05 [3.564e+05, 4.049e+05] | 4 | 3.130e+05 [2.836e+05, 3.416e+05] |
| **RTOP** | Caudate Contra | 11 | 3.483e+05 [3.257e+05, 4.550e+05] | 2 | 3.378e+05 [3.013e+05, 3.744e+05] | 4 | 3.039e+05 [2.697e+05, 3.436e+05] |
| **RTOP** | Putamen Ipsi | 11 | 3.851e+05 [2.998e+05, 4.119e+05] | 2 | 4.625e+05 [4.442e+05, 4.809e+05] | 4 | 3.556e+05 [2.986e+05, 4.148e+05] |
| **RTOP** | Putamen Contra | 11 | 4.350e+05 [3.565e+05, 4.973e+05] | 2 | 4.038e+05 [3.937e+05, 4.138e+05] | 4 | 3.418e+05 [2.656e+05, 4.218e+05] |
| **RTOP** | Thalamus Ipsi | 11 | 4.597e+05 [2.950e+05, 5.355e+05] | 2 | 5.931e+05 [5.866e+05, 5.996e+05] | 4 | 4.135e+05 [3.778e+05, 4.565e+05] |
| **RTOP** | Thalamus Contra | 11 | 4.789e+05 [4.426e+05, 5.195e+05] | 2 | 5.419e+05 [5.101e+05, 5.736e+05] | 4 | 3.985e+05 [3.671e+05, 4.265e+05] |
| **RTOP** | Cerebellar WM Ipsi | 11 | 8.937e+05 [7.017e+05, 9.941e+05] | 2 | 8.906e+05 [8.503e+05, 9.309e+05] | 5 | 8.290e+05 [8.261e+05, 9.064e+05] |
| **RTOP** | Cerebellar WM Contra | 11 | 8.550e+05 [6.994e+05, 9.743e+05] | 2 | 8.858e+05 [8.573e+05, 9.143e+05] | 5 | 9.375e+05 [8.254e+05, 9.510e+05] |
| **RTOP** | Cerebellar GM Ipsi | 11 | 4.145e+05 [3.689e+05, 4.748e+05] | 2 | 3.834e+05 [3.826e+05, 3.841e+05] | 5 | 3.900e+05 [3.195e+05, 3.950e+05] |
| **RTOP** | Cerebellar GM Contra | 11 | 4.294e+05 [4.024e+05, 4.659e+05] | 2 | 4.082e+05 [3.932e+05, 4.233e+05] | 5 | 3.939e+05 [3.919e+05, 4.051e+05] |
| **RTAP** | Corpus callosum | 10 | 1.078e+04 [7828.5, 1.195e+04] | 2 | 1.220e+04 [1.049e+04, 1.390e+04] | 5 | 1.190e+04 [9396.1, 1.214e+04] |
| **RTAP** | CST Ipsi | 9 | 1.025e+04 [9792.0, 1.493e+04] | 2 | 1.466e+04 [1.381e+04, 1.552e+04] | 4 | 1.153e+04 [9462.9, 1.306e+04] |
| **RTAP** | CST Contra | 11 | 1.531e+04 [1.203e+04, 1.600e+04] | 1 | 1.433e+04 [—] | 4 | 1.311e+04 [1.113e+04, 1.421e+04] |
| **RTAP** | Caudate Ipsi | 11 | 4647.5 [3687.1, 5266.0] | 2 | 5319.5 [5051.8, 5587.2] | 4 | 4623.9 [4230.4, 4932.5] |
| **RTAP** | Caudate Contra | 11 | 5084.0 [4753.0, 6083.7] | 2 | 4927.2 [4607.1, 5247.4] | 4 | 4481.0 [4037.7, 4931.1] |
| **RTAP** | Putamen Ipsi | 11 | 5316.3 [4347.5, 5714.4] | 2 | 5938.3 [5782.0, 6094.6] | 4 | 5114.7 [4511.5, 5633.7] |
| **RTAP** | Putamen Contra | 11 | 5719.7 [4957.2, 6151.7] | 2 | 5274.8 [5068.5, 5481.0] | 4 | 4756.0 [3927.1, 5559.3] |
| **RTAP** | Thalamus Ipsi | 11 | 6432.2 [4475.8, 7186.3] | 2 | 7855.3 [7819.5, 7891.0] | 4 | 6048.7 [5614.4, 6432.4] |
| **RTAP** | Thalamus Contra | 11 | 6715.0 [6204.4, 7090.5] | 2 | 7342.1 [7030.2, 7654.1] | 4 | 5893.8 [5494.8, 6142.5] |
| **RTAP** | Cerebellar WM Ipsi | 11 | 1.196e+04 [9445.3, 1.280e+04] | 2 | 1.071e+04 [1.049e+04, 1.093e+04] | 5 | 1.073e+04 [1.033e+04, 1.074e+04] |
| **RTAP** | Cerebellar WM Contra | 11 | 1.129e+04 [9426.1, 1.251e+04] | 2 | 1.108e+04 [1.102e+04, 1.115e+04] | 5 | 1.096e+04 [1.086e+04, 1.177e+04] |
| **RTAP** | Cerebellar GM Ipsi | 11 | 5718.2 [5415.2, 5967.4] | 2 | 5075.3 [5041.6, 5109.1] | 5 | 5225.2 [4509.4, 5377.7] |
| **RTAP** | Cerebellar GM Contra | 11 | 5416.3 [5205.5, 5980.2] | 2 | 5915.7 [5772.3, 6059.2] | 5 | 5324.7 [5315.0, 5355.8] |
| **RTPP** | Corpus callosum | 10 | 48.161 [46.971, 49.901] | 2 | 49.300 [48.057, 50.543] | 5 | 48.662 [46.442, 49.882] |
| **RTPP** | CST Ipsi | 9 | 52.131 [51.519, 53.118] | 2 | 54.113 [52.872, 55.354] | 4 | 50.837 [49.979, 51.565] |
| **RTPP** | CST Contra | 11 | 53.200 [51.516, 53.803] | 1 | 55.377 [—] | 4 | 52.180 [50.318, 53.846] |
| **RTPP** | Caudate Ipsi | 11 | 56.681 [47.942, 58.452] | 2 | 58.235 [56.725, 59.745] | 4 | 57.135 [55.600, 58.307] |
| **RTPP** | Caudate Contra | 11 | 58.353 [55.115, 59.326] | 2 | 54.527 [51.135, 57.920] | 4 | 57.171 [53.760, 59.777] |
| **RTPP** | Putamen Ipsi | 11 | 58.108 [54.932, 60.207] | 2 | 64.063 [63.856, 64.271] | 4 | 58.400 [55.710, 60.519] |
| **RTPP** | Putamen Contra | 11 | 63.337 [58.693, 64.479] | 2 | 59.333 [57.477, 61.190] | 4 | 57.120 [50.958, 63.178] |
| **RTPP** | Thalamus Ipsi | 11 | 55.097 [52.169, 56.294] | 2 | 59.008 [58.153, 59.862] | 4 | 54.647 [53.718, 55.904] |
| **RTPP** | Thalamus Contra | 11 | 56.747 [55.749, 57.781] | 2 | 56.439 [55.088, 57.790] | 4 | 54.059 [51.706, 56.653] |
| **RTPP** | Cerebellar WM Ipsi | 11 | 60.112 [59.433, 60.815] | 2 | 62.672 [62.125, 63.219] | 5 | 59.377 [58.959, 60.261] |
| **RTPP** | Cerebellar WM Contra | 11 | 59.979 [59.640, 61.864] | 2 | 61.312 [60.303, 62.321] | 5 | 59.722 [59.564, 60.132] |
| **RTPP** | Cerebellar GM Ipsi | 11 | 62.621 [60.685, 64.598] | 2 | 60.212 [59.839, 60.585] | 5 | 61.051 [57.072, 62.186] |
| **RTPP** | Cerebellar GM Contra | 11 | 62.749 [60.568, 63.901] | 2 | 59.496 [59.146, 59.846] | 5 | 61.560 [61.184, 62.655] |
| **FA** | Corpus callosum | 10 | 0.580 [0.508, 0.592] | 2 | 0.564 [0.522, 0.605] | 5 | 0.579 [0.483, 0.587] |
| **FA** | CST Ipsi | 9 | 0.470 [0.407, 0.584] | 2 | 0.577 [0.535, 0.620] | 4 | 0.524 [0.477, 0.566] |
| **FA** | CST Contra | 11 | 0.602 [0.530, 0.614] | 1 | 0.555 [—] | 4 | 0.553 [0.505, 0.575] |
| **FA** | Caudate Ipsi | 11 | 0.218 [0.201, 0.233] | 2 | 0.218 [0.204, 0.231] | 4 | 0.206 [0.168, 0.242] |
| **FA** | Caudate Contra | 11 | 0.223 [0.198, 0.233] | 2 | 0.261 [0.242, 0.279] | 4 | 0.177 [0.172, 0.185] |
| **FA** | Putamen Ipsi | 11 | 0.268 [0.229, 0.300] | 2 | 0.274 [0.248, 0.301] | 4 | 0.249 [0.226, 0.258] |
| **FA** | Putamen Contra | 11 | 0.240 [0.230, 0.286] | 2 | 0.308 [0.271, 0.344] | 4 | 0.232 [0.220, 0.239] |
| **FA** | Thalamus Ipsi | 11 | 0.299 [0.270, 0.320] | 2 | 0.312 [0.308, 0.315] | 4 | 0.288 [0.281, 0.291] |
| **FA** | Thalamus Contra | 11 | 0.300 [0.289, 0.320] | 2 | 0.330 [0.326, 0.335] | 4 | 0.290 [0.274, 0.300] |
| **FA** | Cerebellar WM Ipsi | 11 | 0.416 [0.380, 0.453] | 2 | 0.420 [0.402, 0.438] | 5 | 0.410 [0.387, 0.417] |
| **FA** | Cerebellar WM Contra | 11 | 0.386 [0.356, 0.453] | 2 | 0.412 [0.403, 0.421] | 5 | 0.425 [0.408, 0.443] |
| **FA** | Cerebellar GM Ipsi | 11 | 0.188 [0.178, 0.194] | 2 | 0.209 [0.200, 0.219] | 5 | 0.183 [0.178, 0.191] |
| **FA** | Cerebellar GM Contra | 11 | 0.190 [0.178, 0.196] | 2 | 0.200 [0.191, 0.209] | 5 | 0.188 [0.182, 0.189] |
| **MD** | Corpus callosum | 10 | 8.252e-04 [8.031e-04, 8.858e-04] | 2 | 8.498e-04 [8.131e-04, 8.865e-04] | 5 | 8.309e-04 [8.088e-04, 8.703e-04] |
| **MD** | CST Ipsi | 9 | 7.759e-04 [7.268e-04, 8.196e-04] | 2 | 7.250e-04 [7.241e-04, 7.258e-04] | 4 | 7.714e-04 [7.563e-04, 7.812e-04] |
| **MD** | CST Contra | 11 | 7.273e-04 [6.850e-04, 7.400e-04] | 1 | 7.053e-04 [—] | 4 | 7.443e-04 [7.088e-04, 8.073e-04] |
| **MD** | Caudate Ipsi | 11 | 8.934e-04 [8.332e-04, 0.001] | 2 | 8.848e-04 [8.043e-04, 9.653e-04] | 4 | 7.913e-04 [7.531e-04, 8.904e-04] |
| **MD** | Caudate Contra | 11 | 7.818e-04 [7.229e-04, 8.462e-04] | 2 | 9.068e-04 [8.093e-04, 0.001] | 4 | 8.278e-04 [7.772e-04, 9.310e-04] |
| **MD** | Putamen Ipsi | 11 | 8.159e-04 [7.795e-04, 9.476e-04] | 2 | 7.461e-04 [7.042e-04, 7.880e-04] | 4 | 7.992e-04 [7.065e-04, 9.364e-04] |
| **MD** | Putamen Contra | 11 | 7.343e-04 [6.843e-04, 7.783e-04] | 2 | 8.095e-04 [7.498e-04, 8.693e-04] | 4 | 8.603e-04 [6.768e-04, 0.001] |
| **MD** | Thalamus Ipsi | 11 | 8.536e-04 [8.144e-04, 0.001] | 2 | 8.139e-04 [7.819e-04, 8.459e-04] | 4 | 8.677e-04 [8.251e-04, 9.098e-04] |
| **MD** | Thalamus Contra | 11 | 8.163e-04 [7.528e-04, 8.606e-04] | 2 | 8.710e-04 [8.165e-04, 9.255e-04] | 4 | 8.818e-04 [8.158e-04, 9.581e-04] |
| **MD** | Cerebellar WM Ipsi | 11 | 6.514e-04 [6.340e-04, 6.797e-04] | 2 | 6.515e-04 [6.366e-04, 6.663e-04] | 5 | 6.846e-04 [6.664e-04, 6.892e-04] |
| **MD** | Cerebellar WM Contra | 11 | 6.568e-04 [6.426e-04, 6.888e-04] | 2 | 6.695e-04 [6.612e-04, 6.777e-04] | 5 | 6.747e-04 [6.619e-04, 6.864e-04] |
| **MD** | Cerebellar GM Ipsi | 11 | 7.667e-04 [7.070e-04, 8.356e-04] | 2 | 8.270e-04 [8.143e-04, 8.397e-04] | 5 | 7.944e-04 [7.805e-04, 9.483e-04] |
| **MD** | Cerebellar GM Contra | 11 | 7.586e-04 [7.188e-04, 8.079e-04] | 2 | 8.397e-04 [8.260e-04, 8.535e-04] | 5 | 7.674e-04 [7.606e-04, 7.977e-04] |

**Note.** Lesion-location stratification is exploratory and descriptive. The multifocal category includes participants with multiple small spatially discrete infarcts for which reliable automated LINDA lesion segmentation was not feasible. Subgroup sizes were small and unequal; therefore, no inferential statistical comparisons were performed. Values are reported as median [interquartile range]. For cells containing a single available observation, the median is reported without an interquartile range.

Cell-specific n values reflect the subject-level median ROI observations available for that metric and ROI; therefore, n may be smaller than the overall lesion-category sample size.
