# Supplementary material for: Characterizing post-stroke changes in brain microstructure using the mean apparent propagator model
Source: Neuroimage Clin. 2026 Oct 21;52:104076. doi: 10.1016/j.nicl.2026.104076 (PMC13722559; doi:10.1016/j.nicl.2026.104076)
Supplement: Supplementary file 6 [file mmc6.docx]

**Supplementary Table S1. Correlations between regional diffusion measures and clinical outcomes in participants with chronic stroke**

Values are Spearman rank correlations (ρ), uncorrected p values, and Benjamini-Hochberg false discovery rate (FDR) q values. BH-FDR correction was applied separately within each clinical outcome across all 65 ROI × metric comparisons (13 regions × 5 diffusion metrics). Analyses used participant-level median diffusion values and were restricted to participants with stroke who had available clinical data. Clinical outcome data were available for 15 participants; effective N was 14 for corpus callosum and contralesional CST and 13 for ipsilesional CST because of finalized ROI-level exclusions. No association survived FDR correction (all q > .05).

**RTOP**

| **Region** | **FGA** | **10MWT Comfortable** | **10MWT Fast** | **Fugl-Meyer UE** | **Fugl-Meyer LE** |
| --- | --- | --- | --- | --- | --- |
| Corpus callosum | ρ=.326; p=.256; q=.642 | ρ=-.297; p=.303; q=.668 | ρ=-.244; p=.401; q=.574 | ρ=.224; p=.441; q=.985 | ρ=.136; p=.643; q=.938 |
| CST Ipsi | ρ=.447; p=.126; q=.642 | ρ=-.462; p=.112; q=.668 | ρ=-.549; p=.052; q=.546 | ρ=-.162; p=.596; q=.985 | ρ=.269; p=.375; q=.938 |
| CST Contra | ρ=.342; p=.231; q=.642 | ρ=-.363; p=.203; q=.668 | ρ=-.407; p=.149; q=.546 | ρ=.211; p=.469; q=.985 | ρ=-.116; p=.693; q=.938 |
| Caudate Ipsi | ρ=.250; p=.369; q=.642 | ρ=-.096; p=.732; q=.821 | ρ=-.311; p=.260; q=.546 | ρ=-.057; p=.840; q=.985 | ρ=.161; p=.567; q=.938 |
| Caudate Contra | ρ=.171; p=.543; q=.693 | ρ=-.136; p=.630; q=.787 | ρ=-.250; p=.369; q=.574 | ρ=.045; p=.874; q=.985 | ρ=.083; p=.768; q=.942 |
| Putamen Ipsi | ρ=.216; p=.440; q=.642 | ρ=-.382; p=.160; q=.668 | ρ=-.300; p=.277; q=.546 | ρ=-.073; p=.795; q=.985 | ρ=-.043; p=.878; q=.942 |
| Putamen Contra | ρ=.155; p=.582; q=.693 | ρ=-.121; p=.666; q=.790 | ρ=-.282; p=.308; q=.557 | ρ=-.265; p=.341; q=.985 | ρ=-.049; p=.863; q=.942 |
| Thalamus Ipsi | **ρ=.516; p=.049; q=.642** | ρ=-.300; p=.277; q=.668 | ρ=-.454; p=.089; q=.546 | ρ=.080; p=.776; q=.985 | ρ=.134; p=.634; q=.938 |
| Thalamus Contra | ρ=.273; p=.325; q=.642 | ρ=-.314; p=.254; q=.668 | ρ=-.225; p=.420; q=.574 | ρ=-.263; p=.344; q=.985 | ρ=-.127; p=.653; q=.938 |
| CB-WM Ipsi | ρ=.288; p=.299; q=.642 | ρ=-.171; p=.541; q=.787 | ρ=-.350; p=.201; q=.546 | ρ=.202; p=.470; q=.985 | ρ=-.141; p=.616; q=.938 |
| CB-WM Contra | ρ=.205; p=.464; q=.642 | ρ=-.193; p=.491; q=.787 | ρ=-.232; p=.405; q=.574 | ρ=-.013; p=.965; q=.985 | ρ=.141; p=.616; q=.938 |
| CB-GM Ipsi | ρ=.140; p=.618; q=.693 | ρ=-.361; p=.187; q=.668 | ρ=-.225; p=.420; q=.574 | ρ=.166; p=.554; q=.985 | ρ=.241; p=.388; q=.938 |
| CB-GM Contra | ρ=.228; p=.413; q=.642 | ρ=-.368; p=.177; q=.668 | ρ=-.304; p=.271; q=.546 | ρ=.113; p=.689; q=.985 | ρ=.318; p=.248; q=.938 |

**RTAP**

| **Region** | **FGA** | **10MWT Comfortable** | **10MWT Fast** | **Fugl-Meyer UE** | **Fugl-Meyer LE** |
| --- | --- | --- | --- | --- | --- |
| Corpus callosum | ρ=.286; p=.322; q=.642 | ρ=-.209; p=.474; q=.787 | ρ=-.222; p=.446; q=.574 | ρ=.253; p=.383; q=.985 | ρ=.279; p=.335; q=.938 |
| CST Ipsi | ρ=.350; p=.242; q=.642 | ρ=-.379; p=.201; q=.668 | ρ=-.505; p=.078; q=.546 | ρ=-.303; p=.315; q=.985 | ρ=.288; p=.340; q=.938 |
| CST Contra | ρ=.276; p=.340; q=.642 | ρ=-.231; p=.427; q=.778 | ρ=-.305; p=.288; q=.551 | ρ=.271; p=.349; q=.985 | ρ=-.221; p=.448; q=.938 |
| Caudate Ipsi | ρ=.284; p=.305; q=.642 | ρ=-.136; p=.630; q=.787 | ρ=-.339; p=.216; q=.546 | ρ=-.014; p=.960; q=.985 | ρ=.206; p=.461; q=.938 |
| Caudate Contra | ρ=.255; p=.359; q=.642 | ρ=-.146; p=.603; q=.787 | ρ=-.346; p=.206; q=.546 | ρ=.082; p=.771; q=.985 | ρ=.143; p=.611; q=.938 |
| Putamen Ipsi | ρ=.297; p=.283; q=.642 | ρ=-.507; p=.054; q=.668 | ρ=-.450; p=.092; q=.546 | ρ=.043; p=.879; q=.985 | ρ=.119; p=.672; q=.938 |
| Putamen Contra | ρ=.151; p=.591; q=.693 | ρ=-.157; p=.576; q=.787 | ρ=-.275; p=.321; q=.564 | ρ=-.248; p=.372; q=.985 | ρ=-.036; p=.898; q=.942 |
| Thalamus Ipsi | ρ=.503; p=.056; q=.642 | ρ=-.304; p=.271; q=.668 | ρ=-.457; p=.087; q=.546 | ρ=.030; p=.914; q=.985 | ρ=.145; p=.607; q=.938 |
| Thalamus Contra | ρ=.234; p=.402; q=.642 | ρ=-.325; p=.237; q=.668 | ρ=-.225; p=.420; q=.574 | ρ=-.306; p=.268; q=.985 | ρ=-.163; p=.562; q=.938 |
| CB-WM Ipsi | ρ=.223; p=.425; q=.642 | ρ=-.161; p=.567; q=.787 | ρ=-.300; p=.277; q=.546 | ρ=.025; p=.929; q=.985 | ρ=-.038; p=.893; q=.942 |
| CB-WM Contra | ρ=.253; p=.362; q=.642 | ρ=-.314; p=.254; q=.668 | ρ=-.246; p=.376; q=.574 | ρ=-.177; p=.528; q=.985 | ρ=.172; p=.540; q=.938 |
| CB-GM Ipsi | ρ=.164; p=.560; q=.693 | ρ=-.439; p=.101; q=.668 | ρ=-.307; p=.265; q=.546 | ρ=.154; p=.584; q=.985 | ρ=.253; p=.363; q=.938 |
| CB-GM Contra | ρ=.349; p=.203; q=.642 | ρ=-.446; p=.095; q=.668 | ρ=-.454; p=.089; q=.546 | ρ=.166; p=.554; q=.985 | ρ=.195; p=.485; q=.938 |

**RTPP**

| **Region** | **FGA** | **10MWT Comfortable** | **10MWT Fast** | **Fugl-Meyer UE** | **Fugl-Meyer LE** |
| --- | --- | --- | --- | --- | --- |
| Corpus callosum | ρ=.148; p=.613; q=.693 | ρ=-.226; p=.436; q=.778 | ρ=-.222; p=.446; q=.574 | ρ=-.026; p=.929; q=.985 | ρ=-.310; p=.281; q=.938 |
| CST Ipsi | ρ=.352; p=.238; q=.642 | ρ=-.176; p=.566; q=.787 | ρ=-.121; p=.694; q=.774 | ρ=.173; p=.571; q=.985 | ρ=-.168; p=.584; q=.938 |
| CST Contra | ρ=.294; p=.308; q=.642 | ρ=-.121; p=.681; q=.790 | ρ=-.112; p=.703; q=.774 | ρ=.128; p=.664; q=.985 | ρ=-.198; p=.496; q=.938 |
| Caudate Ipsi | ρ=.230; p=.410; q=.642 | ρ=-.136; p=.630; q=.787 | ρ=-.207; p=.459; q=.574 | ρ=-.011; p=.970; q=.985 | ρ=.206; p=.461; q=.938 |
| Caudate Contra | ρ=.072; p=.799; q=.851 | ρ=-.125; p=.657; q=.790 | ρ=-.050; p=.860; q=.869 | ρ=.059; p=.835; q=.985 | ρ=.099; p=.724; q=.942 |
| Putamen Ipsi | ρ=.261; p=.348; q=.642 | ρ=-.404; p=.136; q=.668 | ρ=-.343; p=.211; q=.546 | ρ=-.084; p=.766; q=.985 | ρ=.043; p=.878; q=.942 |
| Putamen Contra | ρ=.253; p=.362; q=.642 | ρ=-.218; p=.435; q=.778 | ρ=-.389; p=.152; q=.546 | ρ=.023; p=.934; q=.985 | ρ=.098; p=.729; q=.942 |
| Thalamus Ipsi | ρ=.438; p=.102; q=.642 | ρ=-.282; p=.308; q=.668 | ρ=-.386; p=.156; q=.546 | ρ=.129; p=.648; q=.985 | ρ=.029; p=.918; q=.948 |
| Thalamus Contra | ρ=.467; p=.079; q=.642 | ρ=-.364; p=.182; q=.668 | ρ=-.386; p=.156; q=.546 | ρ=.086; p=.761; q=.985 | ρ=.114; p=.686; q=.938 |
| CB-WM Ipsi | ρ=.217; p=.436; q=.642 | ρ=-.014; p=.960; q=.985 | ρ=-.057; p=.840; q=.866 | ρ=.157; p=.576; q=.985 | ρ=-.288; p=.299; q=.938 |
| CB-WM Contra | ρ=.207; p=.460; q=.642 | ρ=-.086; p=.761; q=.839 | ρ=-.236; p=.398; q=.574 | ρ=.159; p=.571; q=.985 | ρ=-.139; p=.621; q=.938 |
| CB-GM Ipsi | ρ=.041; p=.884; q=.926 | ρ=-.079; p=.781; q=.846 | ρ=-.046; p=.869; q=.869 | ρ=.209; p=.454; q=.985 | ρ=.011; p=.969; q=.969 |
| CB-GM Contra | ρ=.149; p=.596; q=.693 | ρ=-.214; p=.443; q=.778 | ρ=-.221; p=.428; q=.574 | ρ=.232; p=.405; q=.985 | ρ=.210; p=.453; q=.938 |

**FA**

| **Region** | **FGA** | **10MWT Comfortable** | **10MWT Fast** | **Fugl-Meyer UE** | **Fugl-Meyer LE** |
| --- | --- | --- | --- | --- | --- |
| Corpus callosum | ρ=.155; p=.597; q=.693 | ρ=-.121; p=.681; q=.790 | ρ=-.090; p=.759; q=.823 | ρ=-.031; p=.917; q=.985 | ρ=.123; p=.676; q=.938 |
| CST Ipsi | ρ=.239; p=.432; q=.642 | ρ=-.198; p=.517; q=.787 | ρ=-.407; p=.168; q=.546 | ρ=-.281; p=.353; q=.985 | ρ=.339; p=.258; q=.938 |
| CST Contra | ρ=.009; p=.976; q=.991 | ρ=-.011; p=.970; q=.985 | ρ=-.143; p=.626; q=.727 | ρ=-.075; p=.799; q=.985 | ρ=-.210; p=.472; q=.938 |
| Caudate Ipsi | ρ=-.079; p=.779; q=.844 | ρ=.104; p=.713; q=.814 | ρ=-.193; p=.491; q=.601 | ρ=-.243; p=.383; q=.985 | ρ=-.253; p=.363; q=.938 |
| Caudate Contra | ρ=.207; p=.460; q=.642 | ρ=-.189; p=.499; q=.787 | ρ=-.375; p=.168; q=.546 | ρ=-.265; p=.341; q=.985 | ρ=.043; p=.878; q=.942 |
| Putamen Ipsi | ρ=-.271; p=.328; q=.642 | ρ=.004; p=.990; q=.990 | ρ=.068; p=.810; q=.849 | ρ=-.300; p=.277; q=.985 | **ρ=-.590; p=.021; q=.938** |
| Putamen Contra | ρ=.020; p=.944; q=.974 | ρ=-.043; p=.879; q=.922 | ρ=-.189; p=.499; q=.601 | ρ=-.350; p=.201; q=.985 | ρ=-.266; p=.338; q=.938 |
| Thalamus Ipsi | ρ=.507; p=.054; q=.642 | ρ=-.368; p=.177; q=.668 | **ρ=-.632; p=.011; q=.546** | ρ=.048; p=.864; q=.985 | ρ=.259; p=.352; q=.938 |
| Thalamus Contra | ρ=.002; p=.995; q=.995 | ρ=-.179; p=.524; q=.787 | ρ=-.075; p=.791; q=.842 | ρ=-.391; p=.149; q=.985 | ρ=-.344; p=.210; q=.938 |
| CB-WM Ipsi | ρ=.295; p=.286; q=.642 | ρ=-.436; p=.104; q=.668 | ρ=-.464; p=.081; q=.546 | ρ=-.025; p=.929; q=.985 | ρ=.051; p=.858; q=.942 |
| CB-WM Contra | ρ=.302; p=.274; q=.642 | ρ=-.386; p=.156; q=.668 | ρ=-.429; p=.111; q=.546 | ρ=-.088; p=.756; q=.985 | ρ=.289; p=.296; q=.938 |
| CB-GM Ipsi | ρ=.259; p=.352; q=.642 | ρ=-.318; p=.248; q=.668 | ρ=-.207; p=.459; q=.574 | ρ=.004; p=.990; q=.990 | ρ=-.212; p=.449; q=.938 |
| CB-GM Contra | ρ=.336; p=.221; q=.642 | ρ=-.146; p=.603; q=.787 | ρ=-.407; p=.132; q=.546 | ρ=-.063; p=.825; q=.985 | ρ=.067; p=.813; q=.942 |

**MD**

| **Region** | **FGA** | **10MWT Comfortable** | **10MWT Fast** | **Fugl-Meyer UE** | **Fugl-Meyer LE** |
| --- | --- | --- | --- | --- | --- |
| Corpus callosum | ρ=-.396; p=.160; q=.642 | ρ=.301; p=.296; q=.668 | ρ=.336; p=.240; q=.546 | ρ=-.330; p=.249; q=.985 | ρ=-.127; p=.665; q=.938 |
| CST Ipsi | ρ=-.463; p=.111; q=.642 | ρ=.407; p=.168; q=.668 | ρ=.478; p=.098; q=.546 | ρ=-.017; p=.957; q=.985 | ρ=-.277; p=.360; q=.938 |
| CST Contra | ρ=-.311; p=.279; q=.642 | ρ=.420; p=.135; q=.668 | ρ=.385; p=.175; q=.546 | ρ=-.209; p=.473; q=.985 | ρ=.149; p=.610; q=.938 |
| Caudate Ipsi | ρ=-.153; p=.587; q=.693 | ρ=.243; p=.383; q=.755 | ρ=.243; p=.383; q=.574 | ρ=-.016; p=.955; q=.985 | ρ=-.231; p=.406; q=.938 |
| Caudate Contra | ρ=-.183; p=.513; q=.681 | ρ=.257; p=.355; q=.744 | ρ=.261; p=.348; q=.574 | ρ=-.143; p=.611; q=.985 | ρ=-.215; p=.441; q=.938 |
| Putamen Ipsi | ρ=-.234; p=.402; q=.642 | ρ=.350; p=.201; q=.668 | ρ=.314; p=.254; q=.546 | ρ=.063; p=.825; q=.985 | ρ=-.136; p=.630; q=.938 |
| Putamen Contra | ρ=-.275; p=.321; q=.642 | ρ=.311; p=.260; q=.668 | ρ=.364; p=.182; q=.546 | ρ=-.045; p=.874; q=.985 | ρ=-.067; p=.813; q=.942 |
| Thalamus Ipsi | **ρ=-.580; p=.023; q=.642** | ρ=.361; p=.187; q=.668 | **ρ=.529; p=.043; q=.546** | ρ=-.232; p=.405; q=.985 | ρ=-.351; p=.200; q=.938 |
| Thalamus Contra | ρ=-.331; p=.229; q=.642 | ρ=.361; p=.187; q=.668 | ρ=.364; p=.182; q=.546 | ρ=-.091; p=.747; q=.985 | ρ=-.042; p=.883; q=.942 |
| CB-WM Ipsi | ρ=-.081; p=.775; q=.844 | ρ=.064; p=.820; q=.874 | ρ=.114; p=.685; q=.774 | ρ=-.097; p=.732; q=.985 | ρ=.376; p=.167; q=.938 |
| CB-WM Contra | ρ=-.261; p=.348; q=.642 | ρ=.157; p=.576; q=.787 | ρ=.282; p=.308; q=.557 | ρ=-.191; p=.495; q=.985 | ρ=-.024; p=.934; q=.948 |
| CB-GM Ipsi | ρ=-.199; p=.476; q=.645 | ρ=.246; p=.376; q=.755 | ρ=.143; p=.612; q=.723 | ρ=-.279; p=.314; q=.985 | ρ=-.049; p=.863; q=.942 |
| CB-GM Contra | ρ=-.216; p=.440; q=.642 | ρ=.293; p=.289; q=.668 | ρ=.336; p=.221; q=.546 | ρ=-.302; p=.274; q=.985 | ρ=-.338; p=.218; q=.938 |

Abbreviations: RTOP, return-to-origin probability; RTAP, return-to-axis probability; RTPP, return-to-plane probability; FA, fractional anisotropy; MD, mean diffusivity; FGA, Functional Gait Assessment; 10MWT, 10-Meter Walk Test; UE, upper extremity; LE, lower extremity; Ipsi, ipsilesional; Contra, contralesional; CB-WM, cerebellar white matter; CB-GM, cerebellar gray matter. Bold entries indicate nominal uncorrected p < .05; none remained significant after FDR correction.
