# Supplementary material for: Characterizing post-stroke changes in brain microstructure using the mean apparent propagator model
Source: Neuroimage Clin. 2026 Oct 21;52:104076. doi: 10.1016/j.nicl.2026.104076 (PMC13722559; doi:10.1016/j.nicl.2026.104076)
Supplement: Supplementary file 7 [file mmc7.docx]

**Table S2.** Clinical Assessment table, displaying time between MRI scan and Clinical Assessments.

| **Participant ID** | **Date of Scan** | **Date of Clinical Assessment** | **Days Between** |
| --- | --- | --- | --- |
| **S01** | 03/05/24 | 03/15/24 | 10 |
| **S02** | 03/11/24 | 03/20/24 | 9 |
| **S03** | 03/12/24 | 03/20/24 | 8 |
| **S04** | 04/10/24 | 04/19/24 | 9 |
| **S05** | 05/29/24 | 07/14/24 | 46 |
| **S06** | 05/07/24 | 05/14/24 | 7 |
| **S08** | 07/26/24 | 08/04/24 | 9 |
| **S09** | 04/09/24 | 04/13/24 | 4 |
| **S12** | 10/03/24 | 10/13/24 | 10 |
| **S13** | 11/14/24 | 11/25/24 | 11 |
| **S14** | 11/19/24 | 11/23/24 | 4 |
| **S15** | 02/27/25 | 03/10/25 | 11 |
| **S16** | 03/06/25 | 03/18/25 | 12 |
| **S17** | 04/21/25 | 04/27/25 | 6 |
| **S18** | 05/02/25 | 05/04/25 | 2 |
| **Overall** | **—** | **—** | **Mean ± SD:**  **10.5 ± 10.2 days** |
| **Maximum interval** | **—** | **—** | **46 days** |
