# Supplementary material for: Characterizing post-stroke changes in brain microstructure using the mean apparent propagator model
Source: Neuroimage Clin. 2026 Oct 21;52:104076. doi: 10.1016/j.nicl.2026.104076 (PMC13722559; doi:10.1016/j.nicl.2026.104076)
Supplement: Supplementary file 8 [file mmc8.docx]

**Supplementary Table S3. Leave-one-out influence analysis of significant regional diffusion findings**

For each of the 54 ROI × metric comparisons significant in the finalized full-cohort analysis (BH-FDR q<0.05 across 65 comparisons), each stroke participant was omitted individually and the Control-versus-Stroke rank-biserial correlation (rᵣᵇ) was recalculated. LOO range gives the minimum–maximum rᵣᵇ across the 18 leave-one-out iterations. Max |Δrᵣᵇ| is the largest absolute deviation from the full-cohort effect. q<0.05 LOO gives the number of 18 iterations that remained significant after separately reapplying BH-FDR across all 65 tests. Directional stability is the primary influence criterion.

| **Metric** | **ROI** | **Full rᵣᵇ** | **Full q** | **LOO rᵣᵇ range** | **Max \|Δrᵣᵇ\|** | **Most influential omitted participant** | **Direction flips** | **q<0.05 LOO** |
| --- | --- | --- | --- | --- | --- | --- | --- | --- |
| RTOP | CB-GM Contra | 0.620 | 0.004 | 0.598 to 0.678 | 0.058 | S12_RV | 0 | 18/18 |
| RTOP | CB-GM Ipsi | 0.579 | 0.006 | 0.554 to 0.659 | 0.080 | S08_RV | 0 | 18/18 |
| RTOP | CB-WM Contra | 0.561 | 0.007 | 0.536 to 0.635 | 0.073 | S19_RV | 0 | 18/18 |
| RTOP | CB-WM Ipsi | 0.544 | 0.008 | 0.517 to 0.635 | 0.091 | S08_RV | 0 | 18/18 |
| RTOP | CST Contra | 0.618 | 0.005 | 0.593 to 0.698 | 0.080 | S04_RV | 0 | 18/18 |
| RTOP | CST Ipsi | 0.846 | <0.001 | 0.835 to 0.902 | 0.057 | S13_RV | 0 | 18/18 |
| RTOP | Caudate Contra | 0.548 | 0.009 | 0.520 to 0.632 | 0.084 | S06_RV | 0 | 18/18 |
| RTOP | Caudate Ipsi | 0.703 | 0.001 | 0.684 to 0.796 | 0.093 | S06_RV | 0 | 18/18 |
| RTOP | Corpus callosum | 0.715 | 0.001 | 0.697 to 0.763 | 0.048 | S03_RV | 0 | 18/18 |
| RTOP | Putamen Ipsi | 0.560 | 0.008 | 0.533 to 0.651 | 0.091 | S06_RV | 0 | 18/18 |
| RTOP | Thalamus Contra | 0.461 | 0.026 | 0.428 to 0.546 | 0.085 | S03_RV | 0 | 18/18 |
| RTAP | CB-GM Contra | 0.550 | 0.008 | 0.523 to 0.622 | 0.073 | S13_RV | 0 | 18/18 |
| RTAP | CB-GM Ipsi | 0.509 | 0.013 | 0.480 to 0.585 | 0.076 | S04_RV | 0 | 18/18 |
| RTAP | CB-WM Contra | 0.708 | 0.001 | 0.690 to 0.796 | 0.088 | S19_RV | 0 | 18/18 |
| RTAP | CB-WM Ipsi | 0.632 | 0.004 | 0.610 to 0.715 | 0.084 | S04_RV | 0 | 18/18 |
| RTAP | CST Contra | 0.566 | 0.008 | 0.537 to 0.642 | 0.076 | S04_RV | 0 | 18/18 |
| RTAP | CST Ipsi | 0.740 | 0.001 | 0.722 to 0.827 | 0.087 | S13_RV | 0 | 18/18 |
| RTAP | Caudate Contra | 0.567 | 0.007 | 0.539 to 0.651 | 0.085 | S06_RV | 0 | 18/18 |
| RTAP | Caudate Ipsi | 0.777 | <0.001 | 0.763 to 0.875 | 0.098 | S06_RV | 0 | 18/18 |
| RTAP | Corpus callosum | 0.765 | <0.001 | 0.750 to 0.816 | 0.051 | S03_RV | 0 | 18/18 |
| RTAP | Putamen Ipsi | 0.449 | 0.029 | 0.414 to 0.533 | 0.084 | S06_RV | 0 | 18/18 |
| RTAP | Thalamus Contra | 0.455 | 0.027 | 0.421 to 0.533 | 0.078 | S03_RV | 0 | 18/18 |
| RTAP | Thalamus Ipsi | 0.418 | 0.041 | 0.382 to 0.493 | 0.075 | S03_RV | 0 | 9/18 |
| RTPP | CB-GM Contra | 0.585 | 0.005 | 0.560 to 0.666 | 0.081 | S08_RV | 0 | 18/18 |
| RTPP | CB-GM Ipsi | 0.585 | 0.005 | 0.560 to 0.666 | 0.081 | S17_RV | 0 | 18/18 |
| RTPP | CST Contra | 0.487 | 0.021 | 0.453 to 0.558 | 0.071 | S03_RV | 0 | 18/18 |
| RTPP | CST Ipsi | 0.761 | 0.001 | 0.744 to 0.857 | 0.096 | S03_RV | 0 | 18/18 |
| RTPP | Caudate Ipsi | 0.567 | 0.007 | 0.539 to 0.645 | 0.078 | S03_RV | 0 | 18/18 |
| RTPP | Corpus callosum | -0.412 | 0.044 | -0.500 to -0.375 | 0.088 | S09_RV | 0 | 7/18 |
| RTPP | Putamen Contra | 0.536 | 0.010 | 0.507 to 0.618 | 0.083 | S06_RV | 0 | 18/18 |
| RTPP | Putamen Ipsi | 0.783 | <0.001 | 0.770 to 0.882 | 0.098 | S06_RV | 0 | 18/18 |
| RTPP | Thalamus Contra | 0.443 | 0.031 | 0.408 to 0.520 | 0.077 | S04_RV | 0 | 13/18 |
| RTPP | Thalamus Ipsi | 0.598 | 0.005 | 0.572 to 0.697 | 0.100 | S03_RV | 0 | 18/18 |
| FA | CB-GM Contra | 0.485 | 0.018 | 0.455 to 0.554 | 0.069 | S04_RV | 0 | 18/18 |
| FA | CB-GM Ipsi | 0.433 | 0.032 | 0.406 to 0.517 | 0.084 | S04_RV | 0 | 18/18 |
| FA | CB-WM Contra | 0.596 | 0.005 | 0.573 to 0.672 | 0.075 | S19_RV | 0 | 18/18 |
| FA | CB-WM Ipsi | 0.602 | 0.005 | 0.579 to 0.690 | 0.088 | S04_RV | 0 | 18/18 |
| FA | CST Ipsi | 0.516 | 0.017 | 0.481 to 0.624 | 0.108 | S13_RV | 0 | 18/18 |
| FA | Corpus callosum | 0.783 | <0.001 | 0.770 to 0.836 | 0.052 | S03_RV | 0 | 18/18 |
| FA | Thalamus Contra | 0.467 | 0.024 | 0.434 to 0.559 | 0.092 | S06_RV | 0 | 18/18 |
| FA | Thalamus Ipsi | 0.616 | 0.005 | 0.592 to 0.691 | 0.075 | S06_RV | 0 | 18/18 |
| MD | CB-GM Contra | -0.626 | 0.004 | -0.690 to -0.604 | 0.065 | S08_RV | 0 | 18/18 |
| MD | CB-GM Ipsi | -0.620 | 0.004 | -0.697 to -0.598 | 0.077 | S04_RV | 0 | 18/18 |
| MD | CB-WM Contra | -0.608 | 0.005 | -0.684 to -0.585 | 0.076 | S08_RV | 0 | 18/18 |
| MD | CB-WM Ipsi | -0.497 | 0.015 | -0.560 to -0.467 | 0.063 | S13_RV | 0 | 18/18 |
| MD | CST Contra | -0.625 | 0.005 | -0.726 to -0.600 | 0.101 | S04_RV | 0 | 18/18 |
| MD | CST Ipsi | -0.895 | <0.001 | -0.977 to -0.887 | 0.083 | S04_RV | 0 | 18/18 |
| MD | Caudate Contra | -0.678 | 0.002 | -0.737 to -0.658 | 0.059 | S08_RV | 0 | 18/18 |
| MD | Caudate Ipsi | -0.721 | 0.001 | -0.796 to -0.704 | 0.075 | S15_RV | 0 | 18/18 |
| MD | Corpus callosum | -0.728 | 0.001 | -0.829 to -0.711 | 0.101 | S04_RV | 0 | 18/18 |
| MD | Putamen Contra | -0.678 | 0.002 | -0.770 to -0.658 | 0.092 | S06_RV | 0 | 18/18 |
| MD | Putamen Ipsi | -0.734 | 0.001 | -0.842 to -0.717 | 0.108 | S06_RV | 0 | 18/18 |
| MD | Thalamus Contra | -0.591 | 0.005 | -0.684 to -0.566 | 0.093 | S04_RV | 0 | 18/18 |
| MD | Thalamus Ipsi | -0.827 | <0.001 | -0.882 to -0.816 | 0.055 | S03_RV | 0 | 18/18 |

Summary: all 54 finalized significant findings retained the same effect direction in every leave-one-out iteration (0/54 direction reversals). Across all 65 comparisons, 64/65 retained direction; the sole unstable-direction comparison was ipsilesional caudate FA, which was non-significant in the full cohort (rᵣᵇ=0.009, q=0.975). RTOP, FA, and MD significant findings remained q<0.05 in all 18 iterations; the least q-stable borderline findings were RTPP corpus callosum (7/18), RTAP ipsilesional thalamus (9/18), and RTPP contralesional thalamus (13/18).
