# Supplementary material for: Characterizing post-stroke changes in brain microstructure using the mean apparent propagator model
Source: Neuroimage Clin. 2026 Oct 21;52:104076. doi: 10.1016/j.nicl.2026.104076 (PMC13722559; doi:10.1016/j.nicl.2026.104076)
Supplement: Supplementary file 9 [file mmc9.docx]

**Supplementary Table S4. Participant-level lesion characteristics and available clinical severity measures.**

| **Participant** | **Hemisphere / pattern** | **Broad anatomical location** | **Lesion volume (mL)** | **FGA** | **10MWT comfortable** | **10MWT fast** | **FM-UE** | **FM-LE** | **Directly overlapped study ROIs** |
| --- | --- | --- | --- | --- | --- | --- | --- | --- | --- |
| S01_RV | Left | Cortical + Subcortical/deep gray + Cerebral white matter + Cerebellar | 7.6 | 15.0 | 8.36 | 7.56 | 56 | 23 | Left CST; Left putamen; Left thalamus; Left cerebellar GM |
| S02_RV | Left | Cortical + Cerebral white matter | 107.9 | 20.0 | 7.55 | 4.42 | 49 | 29 | Corpus callosum; Left CST |
| S03_RV | Right | Brainstem | 0.5 | 22.0 | 6.95 | 4.85 | 52 | 28 | Right CST |
| S04_RV | Left | Cortical + Cerebral white matter | 8.2 | 27.0 | 5.04 | 3.87 | 59 | 28 | None |
| S05_RV | Right | Cortical + Subcortical/deep gray + Cerebral white matter | 189.3 | 18.0 | 6.25 | 6.01 | 20 | 26 | Corpus callosum; Right CST; Right caudate; Right putamen; Right thalamus |
| S06_RV | Right | Cortical + Cerebral white matter | 1.5 | 19.0 | 5.05 | 4.29 | 47 | 28 | None |
| S07_RV | Right | Cortical + Cerebral white matter | 82.6 | NR | NR | NR | NR | NR | Corpus callosum; Right CST; Right caudate; Right putamen |
| S08_RV | Left | Cortical + Subcortical/deep gray + Cerebral white matter + Cerebellar | 6.1 | 27.0 | 5.22 | 3.85 | 62 | 30 | Left caudate; Left putamen; Left cerebellar GM |
| S09_RV | Left | Cortical + Subcortical/deep gray + Cerebral white matter | 20.7 | 22.0 | 5.14 | 4.33 | 60 | 29 | Corpus callosum; Left CST; Left putamen |
| S12_RV | Left | Cortical + Cerebral white matter + Cerebellar | 31.9 | 19.0 | 7.83 | 4.83 | 53 | 27 | Left cerebellar GM |
| S13_RV | Bilateral | Cortical | 1.2 | 19.0 | 6.56 | 4.74 | 27 | 26 | None |
| S14_RV | Left | Cortical + Cerebral white matter | 7.9 | 21.0 | 7.33 | 4.69 | 9 | 28 | None |
| S15_RV | Multifocal / unknown | Distributed/multifocal microlesions; quantitative mask unavailable | N/A | 13.0 | 8.22 | 5.89 | 57 | 27 | Unknown |
| S16_RV | Multifocal / unknown | Distributed/multifocal microlesions; quantitative mask unavailable | N/A | 12.0 | 10.79 | 9.54 | 57 | 20 | Unknown |
| S17_RV | Left | Cortical + Subcortical/deep gray + Cerebral white matter | 24.3 | 8.0 | 56.32 | 49.60 | 8 | 14 | Left CST; Left putamen |
| S18_RV | Multifocal / unknown | Distributed/multifocal microlesions; quantitative mask unavailable | N/A | 12.0 | 7.20 | 7.15 | 15 | 30 | Unknown |
| S19_RV | Multifocal / unknown | Distributed/multifocal microlesions; quantitative mask unavailable | N/A | NR | NR | NR | NR | NR | Unknown |
| S20_RV | Multifocal / unknown | Distributed/multifocal microlesions; quantitative mask unavailable | N/A | NR | NR | NR | NR | NR | Unknown |

**Note.** FGA = Functional Gait Assessment; 10MWT = 10-Meter Walk Test; FM-UE = Fugl-Meyer upper extremity; FM-LE = Fugl-Meyer lower extremity. Lesion volume was calculated only when a reliable quantitative lesion mask was available. S15_RV, S16_RV, S18_RV, S19_RV, and S20_RV had multiple small/microlesions without reliable quantitative segmentation; lesion volume and direct ROI-overlap status are therefore reported as unavailable/unknown rather than zero. NR = not reported in the supplied analysis-ready participant table.
